# Identification, Functional Characterization, and Pharmacological Analysis of Two Sulfakinin Receptors in the Medically-Important Insect *Rhodnius prolixus*

**DOI:** 10.1038/s41598-019-49790-x

**Published:** 2019-09-17

**Authors:** Mark Bloom, Angela B. Lange, Ian Orchard

**Affiliations:** Department of Biology, University of Toronto Mississauga, Mississauga, ON L5L 1C6 Canada

**Keywords:** Neurophysiology, Extracellular signalling molecules

## Abstract

The chordate gastrin/cholecystokinin and ecdysozoan sulfakinin (SK)-signaling systems are functionally and structurally homologous. In the present study, we isolated the cDNA sequences encoding the SK receptors in *Rhodnius prolixus* (Rhopr-SKR-1 and Rhopr-SKR-2). The Rhopr-SKRs have been functionally characterized and their intracellular signaling pathways analysed via a functional receptor assay. Both Rhopr-SKRs are exclusively activated via the two native *R. prolixus* sulfakinins, Rhopr-SK-1 and Rhopr-SK-2, but not via nonsulfated Rhopr-SK-1. The Rhopr-SKRs are each linked to the intracellular Ca^2+^ second messenger pathway, and not to the cyclic AMP pathway. Spatial transcript expression analyses revealed that each Rhopr-SKR is predominantly expressed in the central nervous system with lower expression throughout peripheral tissues. The critical importance of the SK-signaling pathway in the blood-feeding behaviour of *R. prolixus* was demonstrated by knockdown of the transcripts for Rhopr-SKs and Rhopr-SKRs, which results in an increase in the mass of blood meal taken. The parasite causing Chagas disease is transmitted to the host after *R. prolixus* has taken a blood meal, and characterization of the SKRs provides further understanding of the coordination of feeding and satiation, and ultimately the transmission of the parasite.

## Introduction

The chordate gastrin/cholecystokinin (G/CCK) and ecdysozoan sulfakinin (SK) – signaling pathways represent evolutionary divergence of a common ancestral system^1^. The SKs therefore are considered functionally and structurally homologous to the G/CCK neuropeptides; the earliest discovered gastrointestinal hormones^2^. Insect SKs, like the G/CCK in chordates, can function as feeding satiety factors. That is to say, once animals have consumed sufficient amounts of food, they become satiated and cease feeding and/or responding to food stimuli^3,4^. The blood-gorging kissing bug, *Rhodnius prolixus* is a vector of Chagas disease with the parasite transmitted to humans following the blood meal^5^. The blood meal is also the trigger for growth, development, and reproduction in *R. prolixus*. Once per instar, *R. prolixus* gorges on a blood meal; they do not take another blood meal until the next instar^6^. In light of the importance of blood-gorging in *R. prolixus* we sought to examine the SK-signaling pathway and test for its possible influence on the size of blood meal consumed.

The sulfakinin prepropeptide typically codes for two SKs in insects^7–17^. They are myotropic neuropeptides in a variety of insects, and influence contractions of the hindgut, foregut, and heart^2,3,18–24^. Sulfakinins have also been linked to satiety; injection of SK reduced food uptake in *R. prolixus*^17^, the red flour beetle *Tribolium castaneum*^25^, the blow fly *Phormia regina*^26^, the German cockroach *Blatella germanica*^20^, and the desert locust *Schistocerca gregaria*^27^. Suppression of the SK transcript via RNA interference (RNAi) leads to the stimulation of food intake in *Gryllus bimaculatus*^28^ and in *T. castaneum*^29^. Knockdown of the two SK receptor transcripts via RNAi in *T. castaneum* also stimulated food intake^25,29^.

With regard to G protein-coupled receptors (GPCRs), there are 2 CCK receptors termed CCK receptors 1 (CCK1R) and 2 (CCK2R). Similarly, insects appear to also possess 2 GPCRs for SKs^18,29,30^. Cholecystokinins may be either sulfated or nonsulfated, leading to variation in their binding affinities to CCK1R and CCK2R. Sulfated CCKs activate CCK1R 500 to 1,000-fold more than the nonsulfated counterpart, whereas both sulfated and nonsulfated CCKs bind to CCK2R in an equivalent fashion^30^. In insects, although the majority of SKs possess a sulfated tyrosyl residue in their characteristic *C*-terminal heptapeptide core sequence D/EYGHMRFamide, nonsulfated SKs have been shown to occur *in vivo*^4,9,31^. However, the insect SKRs only appear to bind sulfated SKs and not the non-sulfated SKs^4,16,21,29^.

Despite many studies on SKs in insects, little emphasis has been placed on the SKRs. Insect SKRs have only been functionally characterized in three species, namely *D. melanogaster*, *T. castaneum*, and *Periplaneta americana*. Other insect SKRs have been annotated and, along with the functionally characterized SKRs, belong to the rhodopsin-like family of GPCRs^16,29,32–36^. Thus far, the intracellular signaling cascades associated with SKRs have only been characterized in *D. melanogaster* and *T. castaneum*, and reports related to the intracellular signaling properties of SKRs have been conflicting^15,29,36^. Zels *et al*. found that *T. castaneum* SKRs resemble the signaling properties of the CCK1Rs in vertebrates, which link to both the Ca^2+^ and cAMP pathways via Gα_q_ and Gα_s_, respectively. Yu *et al*. reported that both *T. castaneum* receptors couple exclusively to the Gα_s_ protein upon activation, stimulating the cAMP pathway^29^, similar to the *D. melanogaster* CCKLR-17D1^35^. Another study though found the calcium signaling associated with this receptor to be pertussis toxin (PTX) insensitive, suggesting the *D. melanogaster* SK receptor CCKLR-17D3 exclusively couples to Gα_q/11_ proteins^16^, thereby resembling Type 2 CCKRs^30^.

In the present study, a functional receptor assay utilizing a human embryonic kidney (HEK)-293 cell line expressing a modified cyclic nucleotide-gated (CNG) channel (HEK293/CNG) was performed to confirm the validity of the two sequenced Rhopr-SK GPCRs, namely Rhopr-SKR-1 and Rhopr-SKR-2, as Rhopr-SK receptors, and to examine the second messenger pathways. Sulfated and nonsulfated Rhopr-SK-1 were tested for the importance of sulfation of the tyrosine residue on ligand binding. Reverse transcriptase quantitative PCR (RT-qPCR) revealed the distribution of the transcripts for Rhopr-SKRs in various tissues in 5^th^ instar *R. prolixus*, thereby identifying target tissues for Rhopr-SKs. Finally, knockdown of the transcripts for Rhopr-SKs and Rhopr-SKRs were also performed to examine the *in vivo* role of SKs on the size of blood meal consumed.

## Results

### Cloning and characterization of Rhopr-SKRs

**Figure 1:**
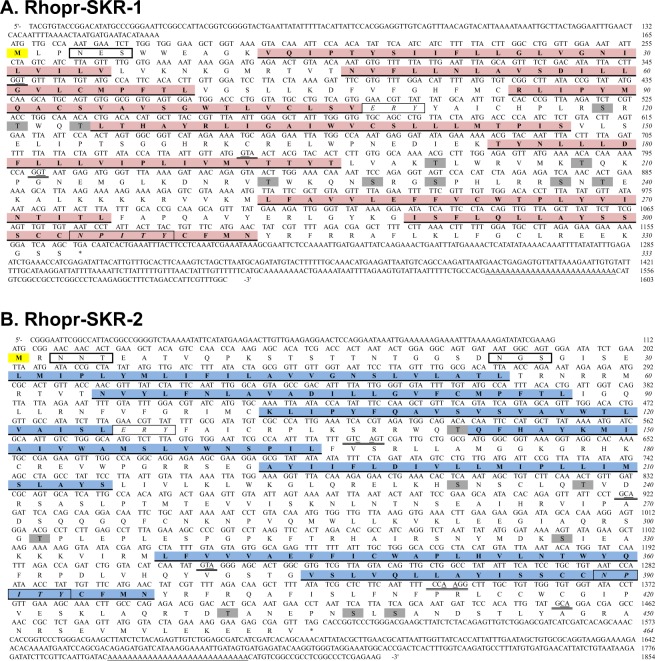
*Rhodnius prolixus* cDNA sequences and the corresponding amino acid sequences of (**A**) Rhopr-SKR-1 and (**B**) Rhopr-SKR-2. The numbers of the nucleotides and amino acids (italicized) are to the right of the sequences. For each receptor, the bolded and highlighted methionine is the translation start site. The predicted *N*-linked glycosylation sites are boxed. The seven predicted hydrophobic transmembrane domains are bolded, highlighted, and underlined. Predicted phosphorylation sites are shaded in grey. Exon boundaries are double-underlined. The ERY and NPITY motifs are italicized and boxed. The 3′ poly(A) tails are underlined.

We successfully isolated cDNA sequences spanning 1603 and 1854 base pairs that encode for Rhopr-SKR-1 and Rhopr-SKR-2, respectively. For Rhopr-SKR-1, the open reading frame (ORF) is 1002 base pairs long, encoding a receptor that is composed of 334 amino acids. This ORF is composed of four exons, which are separated by three introns. A poly(A) tail is present in the 3′UTR. Two stop codons are present in the 5′UTR upstream of the start codon, suggesting that the receptor’s ORF is complete. Phosphorylation is predicted at ten amino acid residues. Rhopr-SKR-1 is predicted to contain an extracellular *N*-terminus, an intracellular *C*-terminus, as well as seven transmembrane domains. *N*-linked glycosylation is predicted at one amino acid residue in the extracellular *N*-terminus (Figs 1A and S1).

For Rhopr-SKR-2, the ORF is 1395 base pairs long, which encodes a SK receptor that is composed of 465 amino acids; this ORF is composed of six exons, which are separated by five introns. A poly(A) tail is present in the 3′UTR. Two stop codons are present in the 5′UTR, upstream of the start codon, suggesting that the receptor’s ORF is complete. Phosphorylation is predicted at eight amino acid residues in its intracellular loops. *N*-linked glycosylation is predicted at two sites at the extracellular *N*-terminus. This GPCR is predicted to contain an extracellular *N*-terminus, as well as an intracellular *C*-terminus, along with seven transmembrane domains (Figs 1B and S2).

Rhopr-SKR-1 and Rhopr-SKR-1 amino acid sequences were aligned via Clustal Omega with a number of their sequenced orthologs from various species, which highlights the high degree of conservation of the transmembrane domains amongst SKRs (Fig. S3). The regions encompassing the seven transmembrane domains of Rhopr-SKR-1 and Rhopr-SKR-2 are highly conserved amongst all of the aligned receptor sequences. The *C*-termini are also highly conserved, whilst the *N*-termini contain little conservation. Three of the predicted phosphorylation sites are conserved amongst the receptors, suggesting their functionality. The *N*-linked glycosylation sites are not conserved amongst the examined receptors (Fig. S3). Rhopr-SKR-1 and Rhopr-SKR-2 display a high degree of similarity, with approximately 43% amino acid identity. Conserved sites that are commonly found in the rhodopsin-like family of GPCRs, such as the intracellular ERY motif (following transmembrane domain III) as well as the NPITY motif (in transmembrane domain VII), are present in both Rhopr-SKR-1 and Rhopr-SKR-2 (Fig. 1).

### Functional receptor assays of Rhopr-SKRs

HEK293/CNG cells were utilized to characterize the Rhopr-SKRs and test whether they naturally function via the intracellular Ca^2+^ (through phospholipase C (PLC)) and/or the cAMP second messenger pathway(s) (see the next section for the second messenger pathways). The PLC pathway in HEK293/CNG cells increases intracellular calcium, which is monitored via luminescence. The CNG channel acts as a biosensor for cAMP, such that an increase in intracellular cAMP levels results in the opening of the CNG channel in the membrane, producing an influx of extracellular calcium into the cell, which is also monitored by luminescence.

**Figure 2:**
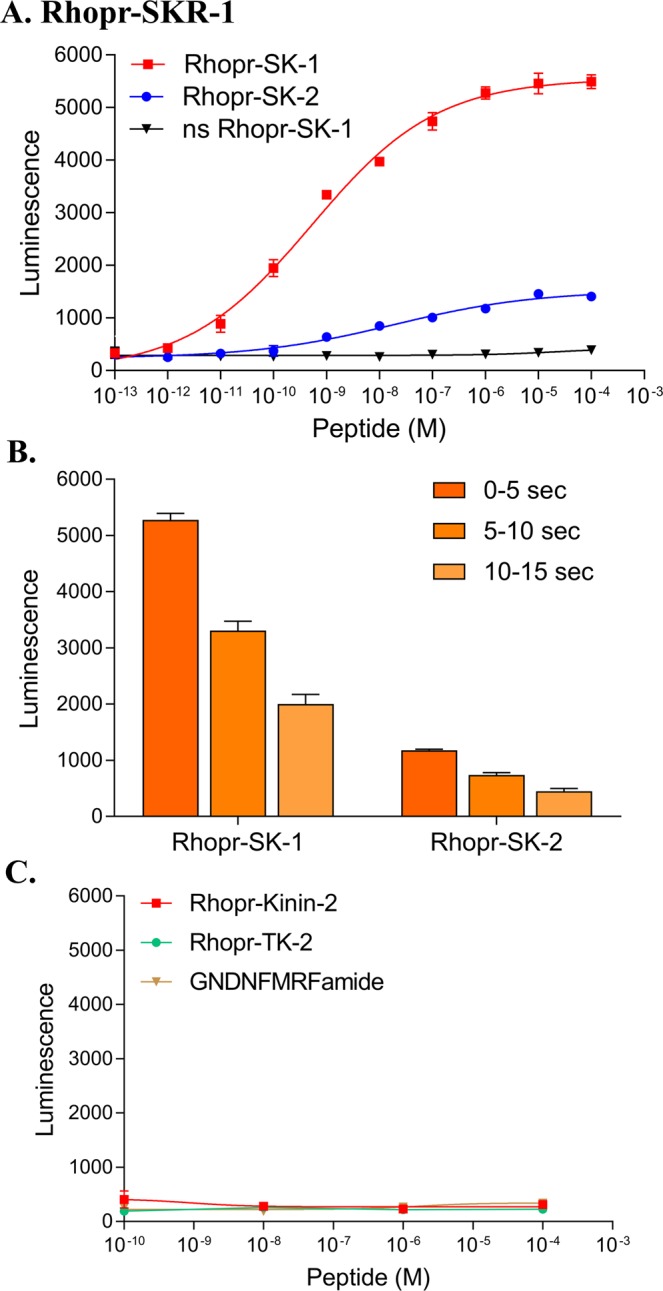
Functional characterization of Rhopr-SKR-1 transiently expressed in HEK293/CNG cells. (**A**) Dose-response curve showing the activation of Rhopr-SKR-1 via Rhopr-SK-1, Rhopr-SK-2, and ns (nonsulfated) Rhopr-SK-1 as ligands (for the first 5-second interval). EC_50_ for Rhopr-SK-1 was 5.32 × 10^−10^ M, whilst the EC_50_ for Rhopr-SK-2 was 8.69 × 10^−9^ M. Nonsulfated Rhopr-SK-1 was unable to elicit a response. (**B**) Kinetics of response activation at 5-second intervals (a total of 15 seconds) at 10^−6^ M for Rhopr-SK-1 and Rhopr-SK-2. (**C**) Kinin-2 (Rhopr-Kinin-2), tachykinin (Rhopr-TK-2), and GNDNFMRFamide showed little to no response. Data points are mean ± standard error of the mean for 3 replicates.

**Figure 3:**
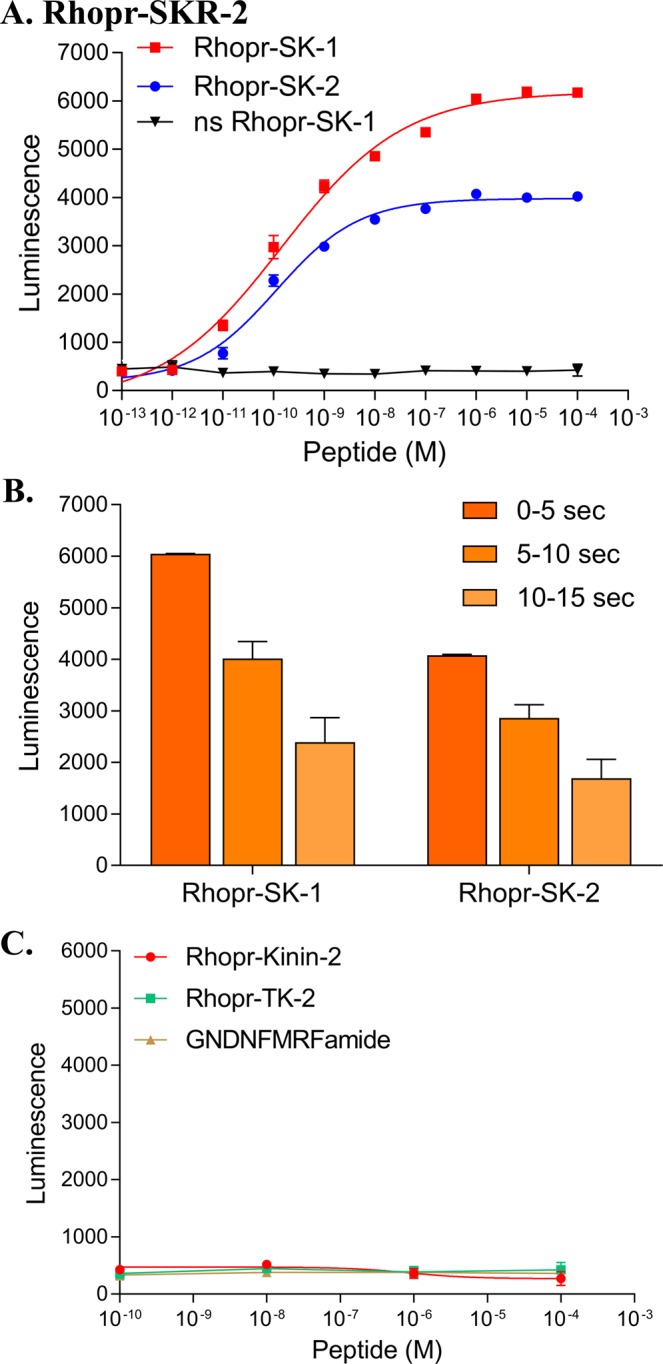
Functional characterization of Rhopr-SKR-2 transiently expressed in HEK293/CNG cells. (**A**) Dose-response curve showing the activation of Rhopr-SKR-2 via Rhopr-SK-1 and Rhopr-SK-2, and ns (nonsulfated) Rhopr-SK-1 as ligands (for the first 5-second interval). EC_50_ for Rhopr-SK-1 was equivalent to 1.44 × 10^−10^ M, whilst the EC_50_ for Rhopr-SK-2 was 1.01 × 10^−10^ M. Nonsulfated Rhopr-SK-1 was unable to elicit a response. (**B**) Kinetics of response activation at 5-second intervals (a total of 15 seconds) at 10^−6^ M for Rhopr-SK-1 and Rhopr-SK-2. (**C**) Kinin-2 (Rhopr-Kinin-2), tachykinin (Rhopr-TK-2), and GNDNFMRFamide showed little to no response. Data points are mean ± standard error of the mean for 3 replicates.

The bioluminescence receptor assay confirmed that the two sequenced Rhopr-SKRs, Rhopr-SKR-1 and Rhopr-SKR-2, are SKRs since they are activated by sulfated Rhopr-SK-1 and sulfated Rhopr-SKR-2 in a dose-dependent manner (Figs 2 and 3). HEK293/CNG cells transfected with empty pIRES2-ZsGreen1 plasmids produce no luminescence in response to the addition of Rhopr-SK-1 or Rhopr-SK-2 (not shown), which demonstrates that the luminescence response is due to the presence and activation of Rhopr-SKRs and not due to endogenous receptors present in the HEK293/CNG cells. For Rhopr-SKR-1 and Rhopr-SKR-2, luminescence responses suggest that Rhopr-SK-2 is a partial, but potent, ligand (Figs 2A and 3A). The luminescence response was maximal at 5 seconds following the addition of the Rhopr-SKs for both Rhopr-SKRs (Figs 2B and 3B). For both Rhopr-SKRs, an extended Rhopr-FMRFamide (GNDNFMRFamide), an *R. prolixus* tachykinin (TK), Rhopr-TK-2, and Rhopr-Kinin-2 did not induce a luminescent response. This indicates that the Rhopr-SKRs are activated by Rhopr-SKs (Figs 2C and 3C). The inability of nonsulfated Rhopr-SK-1 to produce luminescence highlights the importance of the sulfated tyrosyl residue in the peptide’s efficacy when binding to either Rhopr-SKR-1 or Rhopr-SKR-2 (Figs 2A and 3A). For Rhopr-SKR-1, the EC_50_s for Rhopr-SK-1 and Rhopr-SK-2 are 5.32 × 10^−10^ M and 8.69 × 10^−9^ M, respectively (Fig. 2A). For Rhopr-SKR-2, the EC_50_s for Rhopr-SK-1 and Rhopr-SK-2 are 1.44 × 10^−10^ M and 1.01 × 10^−10^ M, respectively (Fig. 3A).

**Figure 4:**
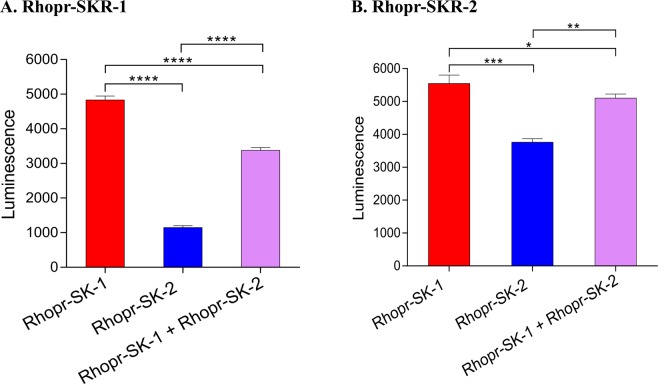
Luminescence response of Rhopr-SK-1 (1 × 10^−6^ M), Rhopr-SK-2 (1 × 10^−6^ M), as well as a mixture of Rhopr-SK-1 and Rhopr-SK-2 (1 × 10^−6^ M) for (**A**) Rhopr-SKR-1 and (**B**) Rhopr-SKR-2. For Rhopr-SKR-1, the observed luminescence was significantly lower for the Rhopr-SK-1 and Rhopr-SK-2 mixture as opposed to Rhopr-SK-1 alone (one way ANOVA followed by Tukey’s Multiple Comparisons Test, ****P < 0.0001). For Rhopr-SKR-2, luminescence was lower for the combination of Rhopr-SK-1 and Rhopr-SK-2 as opposed to Rhopr-SK-1 alone (one way ANOVA followed by Tukey’s Multiple Comparisons Test, *P* < 0.05), and as opposed to Rhopr-SK-2 alone (one way ANOVA followed by Tukey’s Multiple Comparisons Test, **P < 0.01). Here, luminescence was lower for Rhopr-SK-2 when compared to Rhopr-SK-1 (one way ANOVA followed by Tukey’s Multiple Comparisons Test, ***P < 0.001). Both receptors were transiently expressed in HEK293/CNG cells. Data points are mean ± standard error of the mean for 3 replicates.

For both Rhopr-SKRs, the combination of Rhopr-SK-1 and Rhopr-SK-2 resulted in a lower luminescence response when compared to Rhopr-SK-1 alone, indicating that Rhopr-SK-2 competes with Rhopr-SK-1 for both Rhopr-SKRs (Fig. 4).

### Second messenger pathways of Rhopr-SKRs

**Figure 5:**
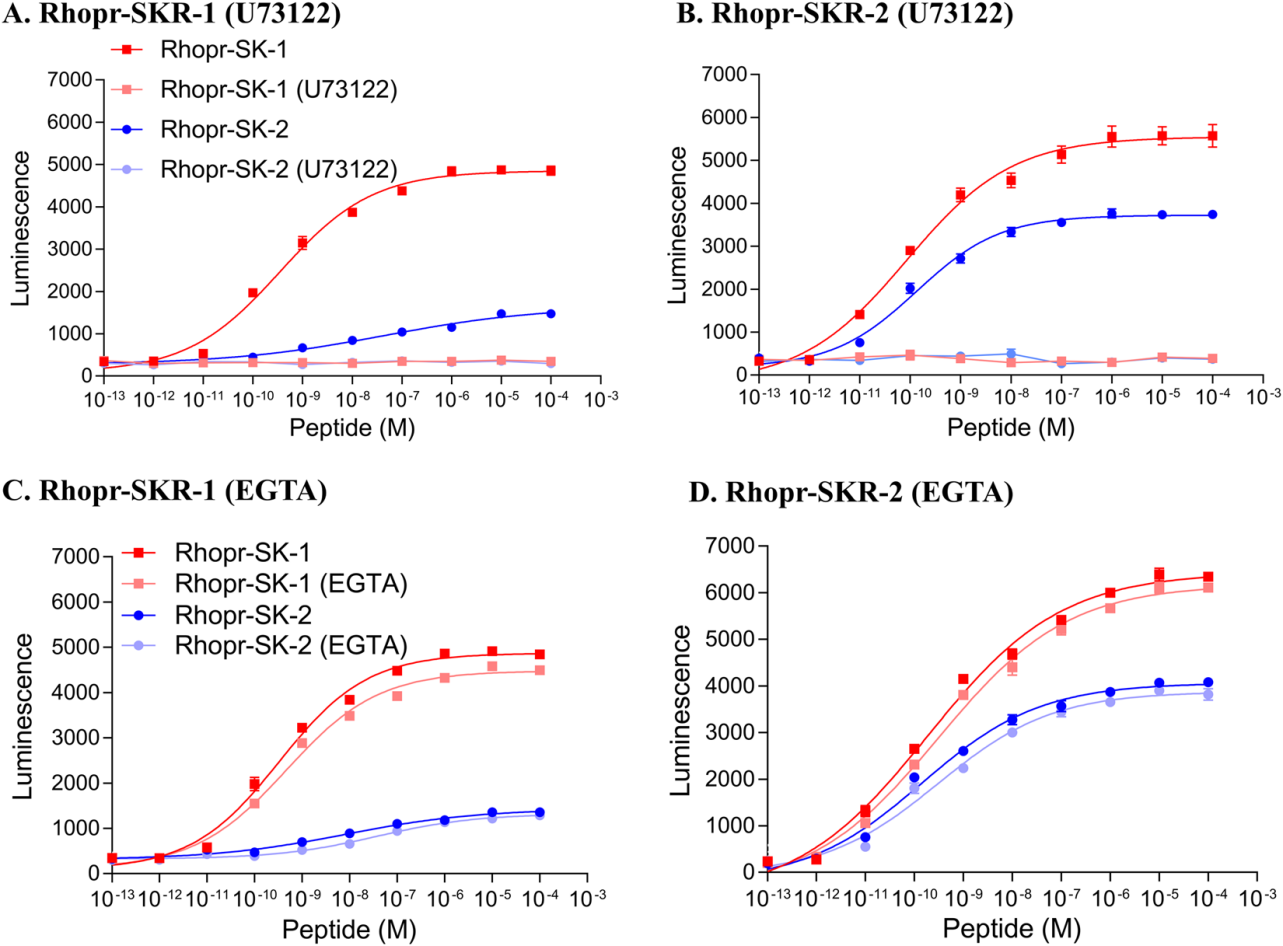
Functional characterizations of Rhopr-SKRs in the presence or absence of the PLC inhibitor, U73122 or in calcium-free media containing EGTA. Namely, (**A**) Rhopr-SKR-1 with U73122, (**B**) Rhopr-SKR-2 with U73122, (**C**) Rhopr-SKR-1 with EGTA, and (**D**) Rhopr-SKR-2 with EGTA. All receptors were transiently expressed in HEK293/CNG cells, with all media containing calcium (in the absence of EGTA). For both Rhopr-SKR-1 and Rhopr-SKR-2, both Rhopr-SK-1 and Rhopr-SK-2 were unable to elicit a response in the presence of 10 μM of U73122 when compared to Rhopr-SK-1 and Rhopr-SK-2 in the absence of U73122. In the absence of U73122 for Rhopr-SKR-1, EC_50_ for Rhopr-SK-1 was 3.17 × 10^−10^ M and 1.33 × 10^−8^ M for Rhopr-SK-2. For Rhopr-SKR-2, EC_50_ for Rhopr-SK-1 was 1.03 × 10^−10^ M and 1.21 × 10^−10^ M for Rhopr-SK-2. In the absence of EGTA for Rhopr-SKR-1, EC_50_ for Rhopr-SK-1 was 2.94 × 10^−10^ M and 6.86 × 10^−9^ M for Rhopr-SK-2. In the presence of EGTA for Rhopr-SKR-1, EC_50_ for Rhopr-SK-1 was 4.40 × 10^−10^ M and 3.02 × 10^−8^ M for Rhopr-SK-2. In the absence of EGTA for Rhopr-SKR-2, EC_50_ for Rhopr-SK-1 was 2.14 × 10^−10^ M and 1.34 × 10^−10^ M for Rhopr-SK-2. In the presence of EGTA for Rhopr-SKR-2, EC_50_ for Rhopr-SK-1 was 3.44 × 10^−10^ M and 3.14 × 10^−10^ M for Rhopr-SK-2. Data points are mean ± standard error of the mean for 3 replicates.

Incubation of HEK293/CNG cells expressing either Rhopr-SKR-1 or Rhopr-SKR-2 in 10 μM of the PLC inhibitor U73122 abolished the luminescence response to Rhopr-SK-1 and Rhopr-SK-2 (Fig. 5A,B) indicating that the receptors are linked to the PLC second messenger pathway.

To test if cAMP might also be a second messenger, HEK293/CNG cells expressing either Rhopr-SKR-1 or Rhopr-SKR-2 were incubated in calcium-free media with the extracellular calcium chelator EGTA (ethylene glycol-bis(β-aminoethyl ether)-N,N,N′,N′-tetraacetic acid). In these cells, if cAMP is elevated by the application of the ligand, then the CNG channels would open, allowing extracellular calcium to enter the cell and be monitored by luminescence. Calcium-free media (containing EGTA to chelate all of the extracellular calcium) would therefore abolish this effect of cAMP. Figure 5C,D shows that calcium-free media containing EGTA does not abolish the luminescence induced by Rhopr-SK-1 and Rhopr-SK-2, indicating that neither Rhopr-SKRs are linked to the cAMP second messenger pathway. Thus, both Rhopr-SKR-1 and Rhopr-SKR-2 solely function via the PLC/IP_3_ pathway, elevating intracellular Ca^2+^ (and not via cAMP). Addition of forskolin as well as dibutyryl-cAMP elicited luminescence responses in cells expressing Rhopr-SKR-1, thereby confirming the presence and functionality of the CNG channels in the HEK293/CNG cells (Fig. S4). Addition of ATP in all conditions elicited the expected positive bioluminescent response, whilst cells in media alone did not elicit luminescence (not shown).

### Spatial expression analyses of Rhopr-SKR-1 and Rhopr-SKR-2 via RT-qPCR

**Figure 6:**
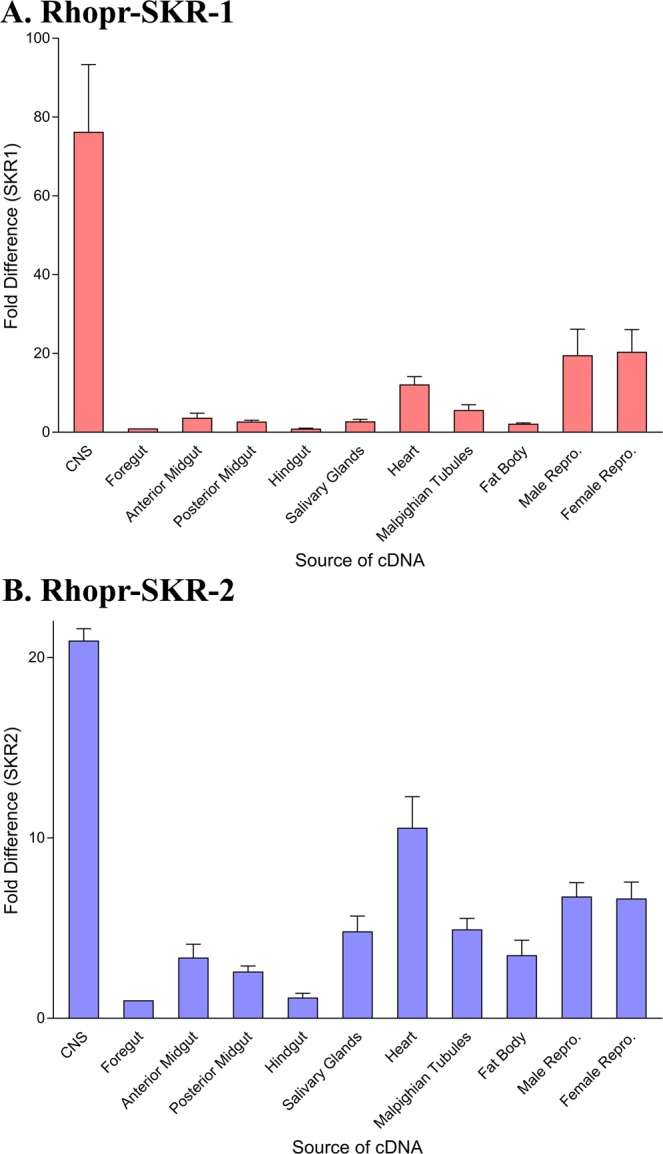
Spatial expressions of the (**A**) Rhopr-SKR-1 and (**B**) Rhopr-SKR-2 transcripts in *Rhodnius prolixus* 5^th^ instars via reverse transcriptase quantitative PCR (RT-qPCR). For each receptor, expression was examined in the CNS, foregut, anterior midgut, posterior midgut, hindgut, salivary glands, heart, Malpighian tubules, fat body, male reproductive system, and female reproductive system. Data points are mean ± standard error of the mean for 3 independent samples.

Transcript expression levels for Rhopr-SKR-1 were highest in the CNS, with lower levels of expression in the male and female reproductive tissues, the heart, Malpighian tubules, salivary glands, fat body, and gut tissues (Fig. 6A). Transcript expression levels for Rhopr-SKR-2 were also highest in the CNS, with relatively lower levels of expression in the heart, salivary glands, Malpighian tubules, as well as the male and female reproductive tissues, gut, and fat body tissues (Fig. 6B).

### Knockdown of Rhopr-SKs and Rhopr-SKRs via double stranded RNA (dsRNA)

RT-qPCR demonstrated the efficiency of Rhopr-SK dsRNA, whereby the Rhopr-SK expression levels were reduced in 5^th^ instar CNS at day 2 post-injection by 96% (n = 3), expressed as the percentage of knockdown in relation to CNS transcripts from insects injected with the double stranded ampicillin resistance gene (dsARG). For the Rhopr-SKR-1 and Rhopr-SKR-2 dsRNA experiment, expression levels of the transcripts at day 2 post-injection were reduced by 93.1% and 96.8%, respectively (n = 3).

**Figure 7:**
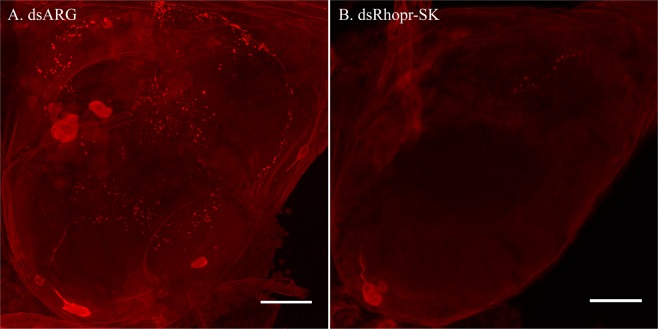
Verification of Rhopr-SK knockdown via immunohistochemical staining of the brain in 5th instar *Rhodnius prolixus*. Insects were injected with the dsRNA of either (**A**) ARG (dsARG) or (**B**) Rhopr-SK (dsRhopr-SK) then dissected and examined two days post-injection. Animals injected with dsARG display bright immunoreactive staining throughout the cells and processes of the brain, whereas Rhopr-SK dsRNA-injected animals display reduced staining throughout the same regions. Scale bars: 100 μm.

Immunohistochemistry was utilized to verify the efficiency of Rhopr-SK dsRNA knockdown *in vivo*. Since the Rhopr-SK transcript codes for both Rhopr-SK-1 and Rhopr-SK-2, the reduction or absence of SK-like immunohistochemical staining in insects pre-injected with Rhopr-SK dsRNA confirms that both Rhopr-SKs have decreased expression in the *R. prolixus* CNS. SK-like staining was drastically reduced in CNS tissues from animals injected with dsRNA (dsRhopr-SK) in relation to insects injected with dsARG (Fig. 7).

Silencing of the Rhopr-SKs led to an increase in the overall intake of the blood meal by 31.4%, compared to control animals injected with dsARG. Moreover, the simultaneous silencing of the Rhopr-SKR-1 and Rhopr-SKR-2 led to an increase in the intake of the blood meal by 31.5%, compared to animals injected with dsARG. Such results demonstrate the role of the Rhopr-SK-signaling pathway in satiety in *R. prolixus in vivo*.

**Figure 8:**
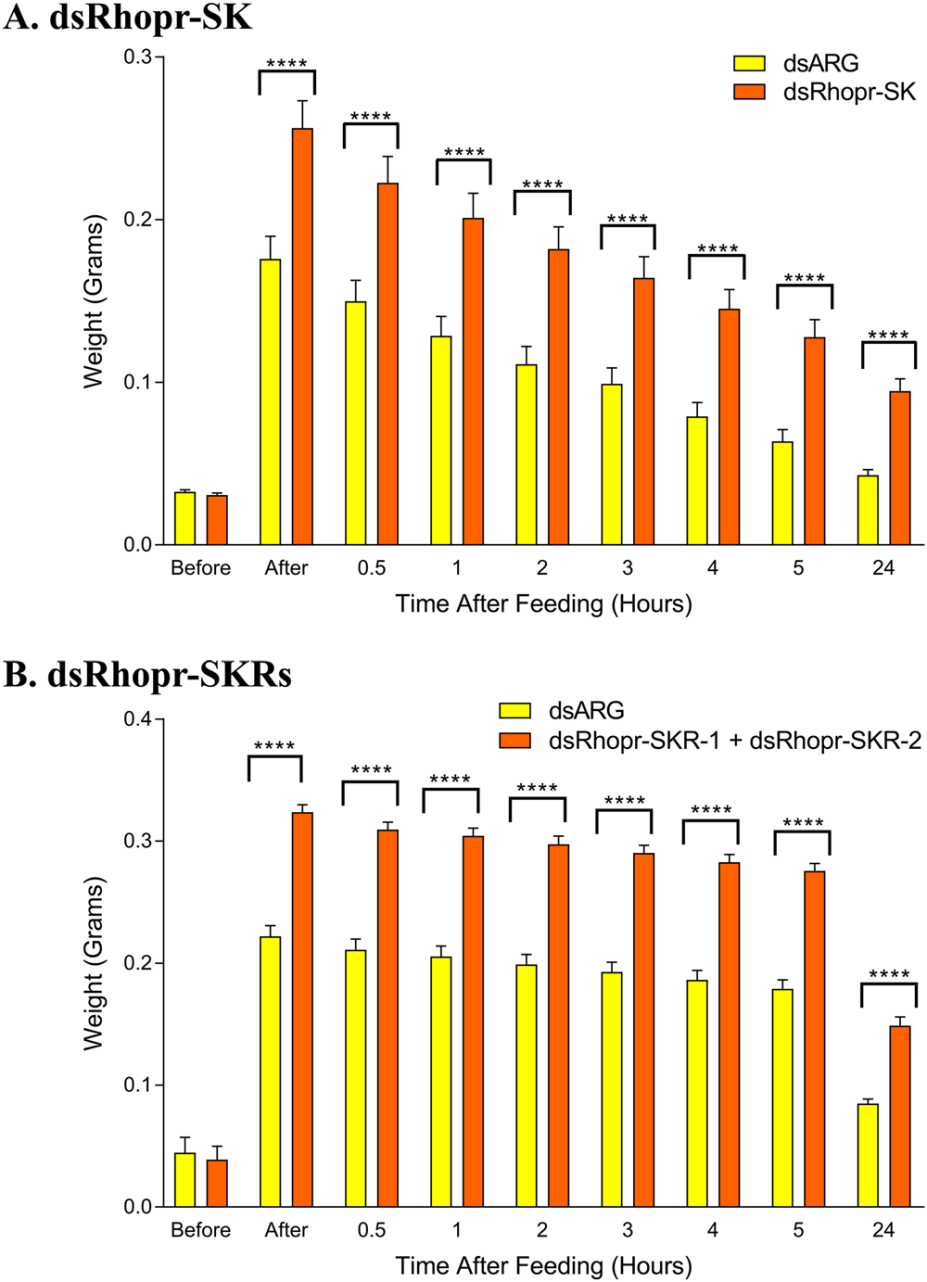
Post-feeding weights of 5^th^ instar *Rhodnius prolixus* previously injected (48 hours earlier) with (**A**) 1 μg of Rhopr-SK dsRNA (dsRhopr-SK) or 1 μg of ARG dsRNA (dsARG); or with (**B**) a mixture of 1 μg of Rhopr-SKR-1 and 1 μg of Rhopr-SKR-2 dsRNA (dsRhopr-SKRs) or 2 μg of ARG dsRNA (dsARG). Post-feeding weights were significantly lower in control insects injected with ARG dsRNA in comparison to insects injected with either Rhopr-SK dsRNA (two way ANOVA followed by Sidak’s Multiple Comparisons Test, ****P < 0.0001) or Rhopr-SKR-1 with Rhopr-SKR-2 dsRNA (two way ANOVA followed by Sidak’s Multiple Comparisons Test, ****P < 0.0001). Histograms are mean ± standard error of the mean for 25 insects (for each of **A**,**B**).

Over the five hours post-feeding, the rate of diuresis (as judged by the loss of weight) was similar between the dsRhopr-SK and dsARG-injected animals, as well as between dsRhopr-SKR-1 + dsRhopr-SKR-2 and dsARG-injected animals, suggesting that Rhopr-SKs and their receptors do not play any direct roles in the rate of diuresis in *R. prolixus* (Fig. 8).

## Discussion

In this study, the full coding sequences for both Rhopr-SKR-1 and Rhopr-SKR-2 were cloned and characterized for the first time in the kissing bug *R. prolixus*. Previously, insect SKRs have only been sequenced in *P. americana*^34^, *T. castaneum*^29^, and *D. melanogaster*^15,35^. The seven hydrophobic transmembrane domains of both Rhopr-SK GPCRs display high sequence similarity to those of other sequenced and predicted SKRs, all of which contain an extracellular *N*-termini and an intracellular *C*-termini^16,25,29,34,36,37^. Conserved signature sequences that are found in the rhodopsin-like family of GPCRs and that are essential for receptor activation are also present in both Rhopr-SKR-1 and Rhopr-SKR-2, including the intracellular ERY motif downstream of transmembrane domain III, as well as the NPITY motif that is within transmembrane domain VII. The amino acid sequences of the Rhopr-SKRs exhibit a high degree of sequence homology with one another, displaying 43% similarity. In relation to their human homologs CCK1R and CCK2R, Rhopr-SKR-1 displays a higher degree of sequence homology with CCK1R (40.5%) than with CCK2R (35.8%), whilst Rhopr-SKR-2 displays an approximately equal degree of sequence homology with both CCK1R and CCK2R (33.5% and 33.4%, respectively).

The signaling properties of both Rhopr-SKR-1 and Rhopr-SKR-2 were analyzed in this study. Both Rhopr-SKR-1 and Rhopr-SKR-2 were activated by sulfated Rhopr-SK-1 and sulfated Rhopr-SK-2 in a dose-dependent manner, indicating that these two peptides are ligands for the receptors. In addition, the results indicate that both Rhopr-SKR-1 and Rhopr-SKR-2 are linked to the intracellular Ca^2+^ pathway via Gα_q_, and do not act via the cAMP pathway. Rhopr-SKRs’ stringent Rhopr-SK agonist requirements for their activation was also demonstrated as members of other families of peptides, Rhopr-Kinin-2, Rhopr-TK-2, and an extended Rhopr-FMRFa, were unable to elicit a response. This study also highlights the importance of the sulfated tyrosyl residue in the efficacy of Rhopr-SKs when binding to either Rhopr-SKR-1 or Rhopr-SKR-2, as nonsulfated Rhopr-SK-1 was unable to activate either of the Rhopr-SKRs. Thus, the sulfated tyrosyl is required for optimal binding and activation of Rhopr-SKRs by Rhopr-SKs.

Although the results of our *in vitro* characterization of the Rhopr-SKRs and their second messenger pathways might not reflect all *in vivo* SK-signaling pathways, they are in line with some of the earlier findings regarding SKRs. Mammalian CCKRs and nematode CCK-like receptors have been typically found to function through the heterotrimeric G proteins of the Gα_q_ family, with only CCK1R also coupling to Gα_s_^30,38–41^. Such findings mainly align with our results, whereby both Rhopr-SKRs were found to couple to Gα_q_-dependent signaling pathways. For Rhopr-SKR-1, Rhopr-SK-1 (EC_50_ = 5.32 × 10^−10^ M) was found to be one order of magnitude more potent than Rhopr-SK-2 (EC_50_ = 8.69 × 10^−9^ M). For Rhopr-SKR-2, the potency of Rhopr-SK-1 (EC_50_ = 1.44 × 10^−10^ M) and Rhopr-SK-2 (EC_50_ = 1.01 × 10^−10^ M) were similar. The dose response curves demonstrate that Rhopr-SK-2 serves as a partial, though potent ligand whereas Rhopr-SK-1 acts as a full, and potent ligand in activating the Rhopr-SK receptors. The unique carboxyl-terminal amino acid sequence of Rhopr-SK-2, whereby the histidine is replaced with a tyrosyl residue in the sequence YGHMRFamide, might explain the partial ligand activity in relation to the highly conserved sequence of Rhopr-SK-1.

The transcript profiles of both Rhopr-SKR-1 and Rhopr-SKR-2 demonstrate that expression for both receptors is highest in the CNS, with lower levels of expression in peripheral tissues. Similar distributions of SKRs were found in *T. castaneum*, where expression levels for both of the transcripts were predominantly in the head region^25,29^. Zels *et al*. have found that both SKR-1 and SKR-2 are mainly present in the brain and optic lobes of *T. castaneum*, with lower expression in the salivary glands, gut, fat body, testes, and ovaries^36^. In humans, CCK1R is predominantly expressed in the alimentary tract, whereas CCK2R is mainly expressed in the brain^42^. The widespread expression of both Rhopr-SKRs in peripheral tissues indicates that Rhopr-SKs might serve as neurohormones that circulate in the hemolymph, influencing a variety of tissues. Mammalian CCKRs have been linked to various physiological processes that include, but are not limited to, the regulation of food intake. In humans, CCK1R has been linked to additional processes, which include stimulating pepsinogen secretion and slowing gastrointestinal motility^43^, whilst CCK2R has been linked to memory, anxiety, and the secretion of gastric acid^44^. Additionally, insect SKs have also been linked to synaptic growth^35^, locomotion, odor preference^45^, and release of digestive enzymes^46–49^. In *P. americana*, RT-qPCR demonstrated the presence of an SKR in the CNS, and immunocytochemistry demonstrated the receptor’s presence in gut membrane fractions^34^. The transcript expression of Rhopr-SKRs in the reproductive tissues in 5^th^ instar *R. prolixus* suggests their potential role in reproductive processes such as egg production as well as spermatogenesis. In the insect *Zophobas atratus*, SK-1 was found to inhibit spontaneous oviduct and ejaculatory duct contractions^24,50^. The presence of the Rhopr-SKRs in the fat body indicates potential involvement of SKs in energy storage and release, and indeed, Slocinska *et al*. found that SK-1 affects the concentrations of proteins, lipids, and glycogen in the larval fat bodies of *Z. atratus*^50^.

Silencing of SKs via RNAi has been linked to stimulation in the intake of food in *G. bimaculatus*^28^ and *T. castaneum*^29^. Additionally, RNAi-induced silencing of each of the transcripts for the two SKRs in *T. castaneum* led an increase of food intake by approximately 20% for SKR1^25^ and 50% for SKR2^29^. Through the use of a feeding assay, we previously demonstrated the role of Rhopr-SKs in the regulation of food intake in *R. prolixus*^17^, whereby insects injected with Rhopr-SK-1 took smaller blood meals. In the present study, the transcript coding for Rhopr-SK-1 and Rhopr-SK-2 as well as the transcripts coding for Rhopr-SKR-1 and Rhopr-SKR-2 were successfully silenced via injection of their dsRNA into 5^th^ instar *R. prolixus*. Silencing expression of the Rhopr-SKs and the Rhopr-SKRs in *R. prolixus* led to an increase in the overall size of the blood meal consumed by 31.4% and 31.5%, respectively.

In conclusion, the cDNAs of Rhopr-SKR-1 and Rhopr-SKR-2 have been cloned and functionally characterized. Knockdown results provide strong evidence that the Rhopr-SK-signaling pathway is involved in the regulation of food intake in the kissing bug *R. prolixus*, particularly in satiation. The wide spatial distribution of the two Rhopr-SKRs highlights their importance in modulating feeding-related processes as well as other potential roles in processes such as energy metabolism and reproduction. The two Rhopr-SKRs, which are activated by both sulfated Rhopr-SKs, function exclusively via the intracellular Ca^2+^ second messenger pathway and not via the cAMP second messenger pathway. These findings regarding the localization and signaling pathways of the Rhopr-SKRs contribute to future studies regarding potential applications of SKs in pest management. *R. prolixus* is the vector of Chagas disease, with the parasite passed on to humans following blood-feeding. Interfering with the Rhopr-SK-signaling pathway would impact blood-feeding and thereby interfere with the transmission of Chagas disease.

## Materials and Methods

### Animals

Male and female 5^th^ instar *R. prolixus* were obtained from a colony raised at the University of Toronto Mississauga. Insects were reared at 50% humidity, 25 °C in incubators and fed defibrinated rabbit blood (Cedarlane Laboratories Inc., Burlington, ON, Canada), once in each instar. All tissues were dissected from unfed 5^th^ instars, 3 to 4 weeks after ecdysis into 5^th^ instars.

### Chemicals

Rhopr-SK-1 (pQFNEY(SO_3_H)GHMRFamide) and Rhopr-SK-2 (GGSDEKFDDY(SO_3_H)GYMRFamide) were custom synthesized by SynPeptide (Shanghai, China) at >95% purity, and the sequences including the sulfation were confirmed by the Advanced Protein Technology Centre (Hospital for Sick Children, Toronto, ON). Rhopr-FMRFamide (GNDNFMRFamide), Rhopr-tachykinin-2 (Rhopr-TK-2) (APSTMGFQGVRamide), and Rhopr-Kinin-2 (AKFSSWGamide) were custom synthesized by GenScript (Piscataway, NJ, USA) at >95% purity. All peptides were reconstituted in double-distilled water into stock solutions at 10^−3^ M. In the physiological assays, stock solutions were stored at −20 °C as 10 μl aliquots until working solutions were made using physiological saline (KCl 8.6 mM, NaCl 150 mM, CaCl_2_ 2 mM, NaHCO_3_ 4 mM, glucose 34 mM, MgCl_2_ 8.5 mM, HEPES 5 mM [pH 7.0]). An anti-perisulfakinin antibody was provided by Jan Veenstra (Bordeaux, France), with its specificity described by Veenstra *et al*.^51^. Goat cyanine dye 3 (Cy3) anti-rabbit (IgG) secondary antibody was purchased from Jackson ImmunoResearch Laboratories, Inc. (West Grove, PA, USA). Both antibodies were stored at −20 °C. Triton X-100, forskolin, dibutyryl cAMP, and U73122 were obtained from Sigma Aldrich, Oakville, ON, Canada.

### Cloning and characterization of the transcripts for Rhopr-SKRs

Sulfakinin receptors 1 and 2, Rhopr-SKR-1 and Rhopr-SKR-2, were isolated and characterized from 5^th^ instar *R. prolixus*. Here, the putative, partial, amino acid sequences for the Rhopr-SKRs, which were previously annotated by Ons *et al*.^52^, were used to search the *R. prolixus* genome and transcriptome assemblies via BLAST^53^. The remaining *in silico* procedures were performed in Geneious v. 4.7.6 (http://www.geneious.com). Using the results, putative sequences of the SK receptors were highlighted in the *R. prolixus* genome. Then, 5′ and 3′ rapid amplification of cDNA ends (RACE), as well as a modified 5′ and 3′ RACE technique, was applied to obtain the full open reading frames (ORFs) of Rhopr-SKR-1 and Rhopr-SKR-2 (Thermo Fisher Scientific, Waltham, MA, USA). A library plasmid DNA (pDNR-LIB plasmid) of the central nervous system (CNS) that was previously prepared by Paluzzi *et al*. was used as a template for the modified 5′ and 3′ RACE PCRs^54^. A variety of gene-specific as well as plasmid-specific forward and reverse primers were utilized for the modified 3′ and 5′ RACE reactions. The primers were used to amplify specific products using a nested PCR approach (Table S1). All reactions were performed using an s1000 thermal cycler (BioRad Laboratories, Mississauga, ON, Canada). The PCR thermal cycler conditions were maintained at an initial 3 minutes at 94 °C, which was followed by 30 cycles of 30 seconds at 94 °C, 30 seconds at an annealing temperature appropriate to the utilized primers, and 60 seconds at 72 °C. The final step consisted of running the samples for 10 minutes at 72 °C. Following each PCR reaction, the resulting amplicon/template cDNA were gel purified using the EZ-10 Spin Column DNA Gel Extraction Kit (Bio Basic Inc., Markham, ON) then used as a template for the following (nested) PCR reaction. Subsequently, the final RACE PCR products were gel purified and ligated onto a pGEM T Vector System (Promega Corporation, Madison, WI, USA). The amplified cDNA-containing vectors were extracted from the cells via the EZ-10 Spin Column Plasmid DNA Miniprep Kit (Bio Basic Inc., Markham, ON), and sequenced at The Hospital for Sick Children’s DNA sequencing facility (Toronto, ON, Canada) or at Eurofins Canada (North York, ON, Canada). To ensure the accuracy of the obtained sequences, sequencing results were confirmed via several independent clones (minimum of 3).

### Sequence analysis of Rhopr-SKRs

The transmembrane domains for the Rhopr-SKRs were predicted via TMHMM server 2.0 (http://www.cbs.dtu.dk/services/TMHMM/). Intron-exon boundaries were found via a BLAST search using the *R. prolixus* genome assembly and confirmed using the online splice site prediction tool (http://www.fruitfly.org/seq_tools/splice.html). NetPhos 3.1 server was used to predict the potential intracellular phosphorylation sites (http://www.cbs.dtu.dk/services/NetPhos/), and NetNGlyc 1.0 server for predicting the potential *N*-linked glycosylation sites (http://www.cbs.dtu.dk/services/NetNGlyc/). Alignments of the orthologous receptor sequences were performed via Clustal Omega (http://www.ebi.ac.uk/Tools/msa/clustalo/). Boxshade 3.21 server (http://www.ch.embnet.org/software/BOX_form.html) was used to obtain a multiple sequence alignment figure.

### Mammalian expression vectors and transfection of Rhopr-SKRs

The ORFs of Rhopr-SKR-1 and Rhopr-SKR-2 were amplified via the Q5 High-Fidelity DNA Polymerase (New England Biolabs, Massachusetts, USA). Using sequence-specific primers, SacI and SacII restriction sites were respectively introduced at the 5′ and 3′ ends of both Rhopr-SKR-1 and Rhopr-SKR-2. Kozak translation initiation sequences (GCCACC) were also included at the 5′ end via the forward Rhopr-SKR primers (Table S2)^55^. The receptors were each cloned into the pIRES2-ZsGreen1 plasmid (Clontech, Mountain View, CA, USA).

An aequorin luminescence assay was utilized in order to measure Ca^2+^ signaling in HEK293/CNG cells stably expressing a modified cyclic nucleotide-gated (CNG) channel^56^. The cells were grown in Dulbecco’s Modified Eagle Medium Nutrient Mixture F12-Ham (DMEM/F-12) (Thermo Fisher Scientific, Waltham, MA, USA), with 1% penicillin/streptomycin, 10% fetal bovine serum (FBS), and 100 μg/mL G418 (Geneticin). The HEK293/CNG cells were consistently grown in vented T75 flasks and inclubated at 37 °C in 5% CO_2_. After reaching approximately 95% confluency, the mammalian cells were transiently co-transfected with the pIRES2-ZsGreen1 expression vector (containing Rhopr-SKR-1 or Rhopr-SKR-2) and the cytoplasmic luminescent reporter aequorin via X-tremeGENE^TM^ HP DNA Transfection Reagent (Roche Applied Science, Penzberg, Germany). Transfections were performed at a 2:1 ratio for the transfection reagent to the expression vectors. Cells transiently transfected with empty pIRES2-ZsGreen1 expression vectors served as a negative control. The cells were then incubated at 37 °C in 5% CO_2_ for 72 hours prior to performing the bioluminescence assay.

### Functional receptor assays

The bioluminescence assay was performed as previously described^57^. At 72 hours post-transfection, the HEK293/CNG cells were harvested from the T75 flasks using a PBS with ethylenediaminetetraacetic acid (EDTA) solution then suspended in a bovine serum albumin (BSA) media (DMEM/F-12 with 1% penicillin/streptomycin and 1% BSA). Prior to the functional assay, the cells were incubated in coelenterazine h (Promega, Madison, WI, USA) to a 5 μM final concentration, and then incubated for 4 hours whilst stirring in the dark. The cells were then diluted 8-fold via BSA media. Rhopr-SK-1 and Rhopr-SK-2 (as well as nonsulfated Rhopr-SK-1) were prepared in BSA media then plated in duplicates on 96-well plates, with 50 μL of the cells then automatically injected into each of these wells via a Perkin Elmer Wallac 1420 Victor2 plate reader (Perkin Elmer, San Diego, CA, USA). The reader was used to measure luminescence response in consecutive five-second intervals (15 seconds in total). An extended Rhopr-FMRFamide (GNDNFMRFamide), tachykinin (Rhopr-TK-2), and Rhopr-Kinin-2 were used as controls to test for specificity. ATP was used as a positive control. HEK293/CNG cells transfected with empty pIRES2-ZsGreen1 vectors served as negative controls.

### Second messenger pathways of Rhopr-SKRs

To test whether cAMP and/or Ca^2+^ are the second messengers for Rhopr-SKR-1 and Rhopr-SKR-2, HEK293/CNG cells were transfected with each receptor, as previously mentioned, then split into cells that were suspended in calcium-free DMEM with the extracellular calcium chelator EGTA (10 μM) or cells treated with the PLC inhibitor U73122 (10 μM) (Sigma Aldrich, Oakville, ON, Canada). U73122 has been previously shown to successfully inhibit the PLC pathway in the HEK293/CNG cell line^56^. HEK293/CNG cells with receptors that work via the Ca^2+^ pathway would be unable to evoke luminescence in the presence of U73122. The modified CNG channel in HEK293/CNG causes extracellular Ca^2+^ to flow into the cell upon an increase in cytoplasmic cAMP levels. Receptors that solely work through the cAMP pathway would be unable to produce a response in calcium-free media with EGTA. Moreover, receptors that work via both Ca^2+^ and cAMP would elicit a response in the presence of either U73122 or EGTA in calcium-free media.

HEK293/CNG cells were used via the same protocol as previously stated. Luminescence was measured in 5-second intervals for a total of 30 seconds. In the first set of experiments, the transfected cells were incubated in 10 μM of U73122 for 10 minutes prior to measuring luminescence in response to the addition of sulfated Rhopr-SK-1 or Rhopr-SK-2. To confirm the viability of the utilized cells, media in 1.5% Triton X-100 served as a positive control. In the second set of experiments, cells were suspended in calcium-free DMEM (Thermo Fisher Scientific, Waltham, MA, USA) with 10 μM of the extracellular calcium chelator EGTA. Here, luminescence was measured in response to sulfated Rhopr-SK-1 or Rhopr-SK-2 in calcium-free DMEM in the presence of EGTA. A batch of cells that was also pre-incubated in calcium-free DMEM with EGTA were then washed, incubated in regular DMEM, and then tested in response to sulfated Rhopr-SK-1 and Rhopr-SKR-2. Forskolin, which is a stimulator of adenylate cyclase, and the cAMP analog dibutyryl cAMP (dbcAMP) were utilized as controls to confirm the expression of functional CNG channels in the HEK293/CNG cells. ATP served as a positive control. GraphPad Prism version 8.00 (https://www.graphpad.com) was used to analyze the bioluminescence results.

### Spatial expression analyses of Rhopr-SKRs

The following tissues were used from 5^th^ instars for the spatial expression analysis: CNS, foregut, anterior midgut, posterior midgut, hindgut, salivary glands, heart, Malpighian tubules, fat body, as well as male and female reproductive tissues. RNeasy Plus Mini Kit (Qiagen, Valencia, CA, USA) was used to extract RNA from the tissues. At least 20 ng of the total RNA was used as a template for the corresponding cDNA synthesis via the iScript Reverse Transcription Supermix for RT-qPCR (BioRad Laboratories Ltd., Mississauga, ON, Canada). The RT-qPCR analyses were performed using an Mx4000 Multiplex Quantitative PCR System (Stratagene, Mississauga, ON, Canada), using SsoFast EvaGreen Supermix with Low ROX (BioRad Laboratories Ltd., Mississauga, ON, Canada). The temperature-cycling profile consisted of an initial denaturation step at 95 °C for 30 seconds, followed by 40 cycles of denaturation (5 seconds at 95 °C) and annealing/extension (24 seconds at 57 °C), which was followed by a melt curve analysis (58 °C-to-95 °C). The melt curve analysis as well as gel electrophoresis were performed to confirm the specificity of the obtained products. A 208 base-pair Rhopr-SKR-1 cDNA fragment was amplified via the forward primer, 5′ GAAGCTGGTAAAGTACAAATTC 3′, and the reverse primer, 5′ CGAAATCTTTTAGTAAGGATCCA 3′. For Rhopr-SKR-2, a 165 base-pair cDNA fragment was amplified using the forward primer, 5′ TCCAAGCTGTTTCAGTATCAGTAG 3′, and the reverse primer, 5′ GCGAATTCCACACTAAAGACA 3′. Primer efficiencies were calculated for the respective target Rhopr-SKR. Three reference genes, namely ribosomal protein 49, β-actin and α-tubulin, were also amplified via the primers previously described by Zandawala *et al*.^57^ (Table S3). The levels of expressed transcripts were quantified via the standard curve method. The delta-delta C_t_ (ΔΔCT) method was applied to calculate the relative expression of the transcripts. The transcript expression levels for the Rhopr-SKRs were normalized via geometric averaging of the transcript levels of the housekeeping/reference genes. Each reaction contained two technical replicates as well as a no template control and a no reverse transcriptase control. Three biological replicates were performed.

### Knockdown of Rhopr-SKs and Rhopr-SKRs transcripts via double stranded RNA (dsRNA)

Rhopr-SK-specific primers containing the T7 RNA polymerase promoter sequence overhang was utilized in generating dsRNA, which was injected into 5^th^ instar *R. prolixus*, thereby silencing the transcript expression of Rhopr-SK, which encodes Rhopr-SK-1 and Rhopr-SK-2. A 23 base pair T7 RNA polymerase promoter sequence overhang (5′ TAATACGACTCACTATAGGGAGA 3′) was conjugated onto the 5′ ends of forward and reverse Rhopr-SK or Rhopr-SKR gene-specific primers. Using pre-amplified Rhopr-SK and Rhopr-SKR transcripts as templates, the appropriate primers containing the T7 overhangs were utilized to create new cDNA templates (Table S4). The new cDNA templates were then utilized for the synthesis of double-stranded RNA (dsRNA) via the T7 Ribomax Express RNAi System (Promega, Madison, WI, USA). The length of the dsRNA was 207 base pairs for Rhopr-SK, 208 base pairs for Rhopr-SKR-1, and 165 base pairs for Rhopr-SKR-2. The dsRNA utilized for the knockdown of Rhopr-SKR-1 and Rhopr-SKR-2 were synthesized separately then combined pre-injection. Following the anesthetisation of *R. prolixus* 5^th^ instars with CO_2_, 1 μg of the Rhopr-SK (or Rhopr-SKR-1 with Rhopr-SKR-2) dsRNA was injected into the thoraces of the insects. All controls were injected with 1 μg of ampicillin resistance gene dsRNA (dsARG), which targets a transcript that is not present in *R. prolixus*.

Prior to the feeding assays, for both of the Rhopr-SK and Rhopr-SKR knockdown experiments, RT-qPCR was performed on insects injected with the appropriate dsRNA at days 2, 4, and 6 post-injection. This was done to determine the most optimal transcript-knockdown day, during which the injected insects would be fed. For the Rhopr-SKR experiments, the primers utilized for the RT-qPCR reactions were the same as the ones previously utilized for spatial expression analyses (Table S3). Five of the injected animals were dissected per experiment, and PureLink RNA Mini Kit (Life Technologies Corporation, Carlsbad, CA, USA) was used to extract RNA from the CNS tissues. Using the extracted RNA, cDNA was synthesized via the iScript Reverse Transcription Supermix (BioRad Laboratories Ltd., Mississauga, ON, Canada). RT-qPCR was performed using the synthesized CNS cDNA as a template, with an annealing temperature of 60 °C, and the same settings as previously described. For both Rhopr-SKs and Rhopr-SKRs, RT-qPCR results revealed that the knockdown of the transcripts was most effective two days post-injection. RT-qPCR was also performed on a subset of the injected insects (5 insects per experiment) on the day of feeding (two days post-injection) to confirm the efficiency of the injected dsRNA on the knockdown of the respective transcripts. Each reaction contained two technical replicates as well as a no template control; three biological replicates were performed.

Two days following the injection, feeding was examined. The animals were weighed prior to feeding, then allowed access to 37 °C defibrinated rabbit blood (Cedarlane Laboratories Inc., Burlington, ON, Canada) for 20 minutes. Following the feeding period, the animals were individually weighed. The animals were then weighed 30 minutes post-feeding, every hour for the first 5 hours post-feeding, and then 24 hours post-feeding, in order to monitor excretion.

For experiments using dsRNA for Rhopr-SKs, immunohistochemistry was performed to visually confirm the knockdown of the Rhopr-SK transcript (and therefore absence of expressed SKs) in CNS tissues. The tissues were dissected from two days post-injection with Rhopr-SK dsRNA, and immunohistochemistry was performed as previously described^17^. Immunohistochemistry was also performed on control insects injected with dsARG.

### Statistical analyses

GraphPad Prism version 8.00 (https://www.graphpad.com) was used to statistically analyse the data. All of the relevant data is available upon request.

## Supplementary information

Supplementary Figures and Tables S1-S4
