# Supplementary material for: Identification, Functional Characterization, and Pharmacological Analysis of Two Sulfakinin Receptors in the Medically-Important Insect Rhodnius prolixus
Source: Sci Rep. 2019 Sep 17;9:13437. doi: 10.1038/s41598-019-49790-x (PMC6748952; doi:10.1038/s41598-019-49790-x)
Supplement: Supplementary file 1 — Supplementary Figures and Tables S1-S4 [file 41598_2019_49790_MOESM1_ESM.pdf]

Identification, Functional Characterization, and Pharmacological Analysis of Two Sulfakinin Receptors in the Medically-Important Insect *Rhodnius prolixus*

Supplementary Figures S1 to S4 and
Supplementary Tables S1 to S4

Mark Bloom
Angela B. Lange
Ian Orchard

Supplementary Figure S1. Predicted membrane topology of Rhopr-SKR-1's seven transmembrane domains in *Rhodnius prolixus* (via TMHMM server 2.0).

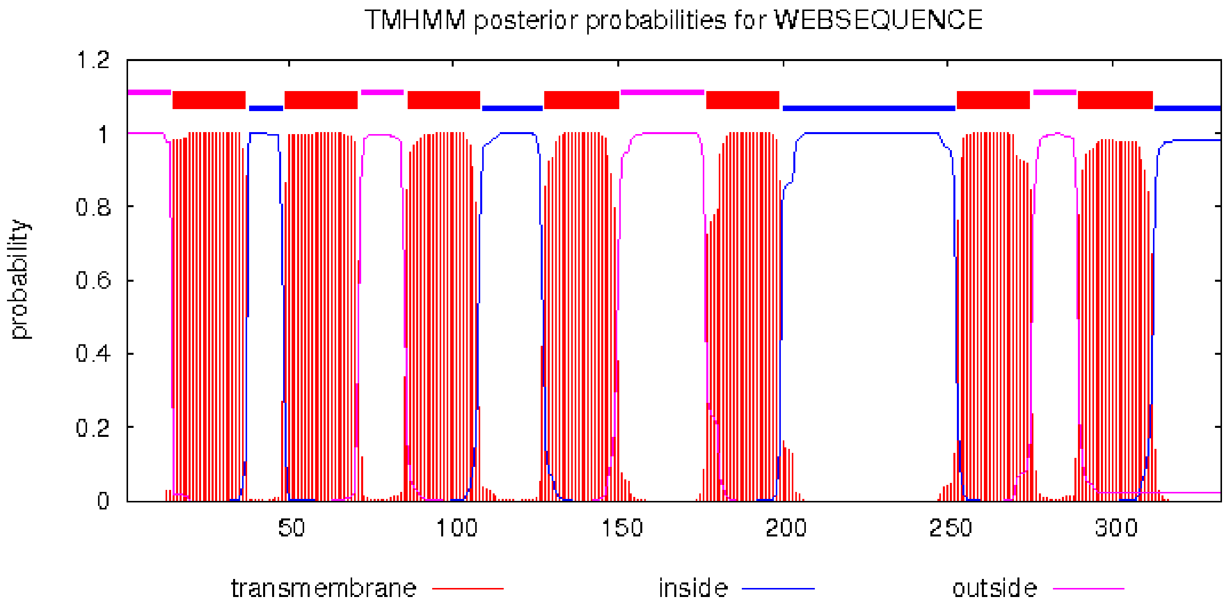
TMHMM posterior probabilities for WEBSEQUENCE
probability
1.2
1
0.8
0.6
0.4
0.2
0
50
100
150
200
250
300
transmembrane
inside
outside

Supplementary Figure S2. Predicted membrane topology of Rhopr-SKR-2’s seven transmembrane domains in *Rhodnius prolixus* (via TMHMM server 2.0).

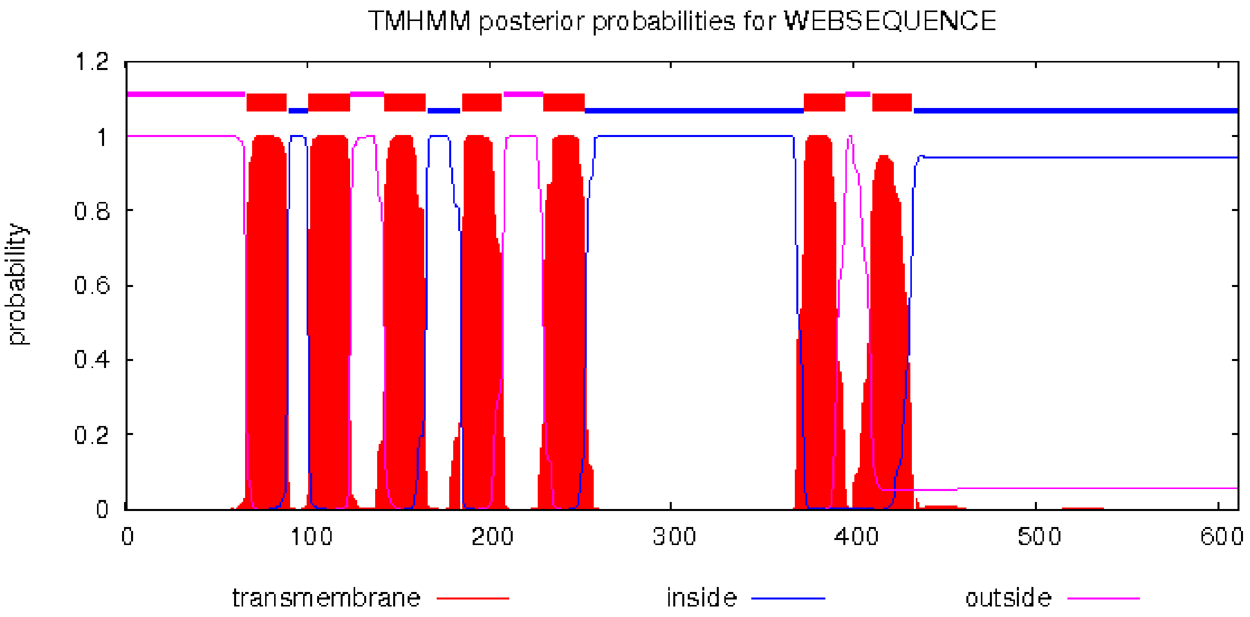
TMHMM posterior probabilities for WEBSEQUENCE
probability
1.2
1
0.8
0.6
0.4
0.2
0
0
100
200
300
400
500
600
transmembrane
inside
outside

Supplementary Figure S3. Amino acid sequence alignment for the *R. prolixus* sulfakinin receptors' transcripts, aligned via Clustal Omega, with homologous receptors. Following the 60% majority rule, identical amino acids are highlighted in black, whilst conserved amino acids are shaded in grey. The seven putative transmembrane domains are indicated via red bars. Dashes were utilized to introduce gaps to maximize homologies. The sequenced used consisted of *Rhodnius prolixus* receptor 1 (GenBank: MK513659) and receptor 2 (GenBank: MK513660), *Tribolium castaneum* sulfakinin receptor 1 (GenBank: AGK29938.1) and receptor 2 (GenBank: XP_972750.1), *Drosophila melanogaster* sulfakinin receptor 1 (GenBank: NP_001097023.1) and receptor 2 (GenBank: NP_001097021.1), *Anopheles gambiae* sulfakinin receptor (GenBank: AAR28375.1), *Periplaneta americana* sulfakinin receptor (GenBank: AAX56942.1), *Nilaparvata lugens* CCK-like receptor (GenBank: XP_022184688.1), *Dendroctonus ponderosae* CCK-like receptor (GenBank: XP_019756917.1), *Nicrophorus vespilloides* CCK-like receptor (GenBank: XP_017773275.1), *Athalia rosae* CCK-like receptor (GenBank: XP_012254014.1), *Neodiprion lecontei* CCK-like receptor (GenBank: XP_015517254.1), *Pediculus humanus* CCK-like receptor (GenBank: XP_002433137.1), *Bactrocera dorsalis* CCK-like receptor (GenBank: XP_011198391.1), *Ooceraea biroi* CCK-like receptor (GenBank: EZA56385.1), *Drosophila persimilis* CCK-like receptor (GenBank: XP_002026780.1), *Homo sapiens* CCK receptor 1 (GenBank: NP_000721.1) and receptor 2 (GenBank: AAA91831.1).

```
Rhodniuspro-SKR1    1 ------------------------------------------------------------
Rhodniuspro-SKR2    1 ------------------------------------------------------------
Tricas-SKR1         1 ------------------------------------------------------------
Tricas-SKR2         1 ------------------------------------------------------------
Drom-SKR1           1 ------------------------------------------------------------
Drom-SKR2           1 MLP-------------RLCADACRQCFAKIARRDTHR-GTRTPYGC---------ADTQS
Anog-SKR            1 ------------------------------------------------------------
Pera-SKR            1 ------------------------------------------------------------
Nill-CCK-like       1 ------------------------------------------------------------
Denp-CCK-like       1 ------------------------------------------------------------
Nicv-CCK-like       1 ------------------------------------------------------------
Athr-CCK-like       1 ------------------------------------------------------------
Neol-CCK-like       1 ------------------------------------------------------------
Pedh-CCK-like       1 ------------------------------------------------------------
Bacd-CCK-like       1 ------------------------------------------------------------
Oocb-CCK-like       1 ------------------------------------------------------------
Drop-CCK-like       1 MLPCCCGELAAAAVGPTCCGSRCRLRATEGANNGASASVLHEGYEARDTPRTVSSVSVLG
Homos-CCKR1         1 ------------------------------------------------------------
Homos-CCKR2         1 ------------------------------------------------------------


Rhodniuspro-SKR1    1 ------------------------------------------------------------
Rhodniuspro-SKR2    1 ------------------------------------------------------------
Tricas-SKR1         1 --------------------------------------------------MSEV----EM
Tricas-SKR2         1 ------------------------------------------------------------
Drom-SKR1           1 ------------------------------------------------------MFNYEE
Drom-SKR2          38 RPKPNFLLREVDEVCCTAASA---SPRLLVLFRD-------HKRASFFGLTIDAFYHYLR
Anog-SKR            1 --------------------------------------------------MTDGPFVYGI
Pera-SKR            1 -----------------------------------------------MEMMDDN----SI
Nill-CCK-like       1 ------------------------------------------------------------
Denp-CCK-like       1 ------------------------------------------------------------
Nicv-CCK-like       1 ------------------------------------------------------------
Athr-CCK-like       1 ------------------------------------------------------------
Neol-CCK-like       1 ------------------------------------------------------------
Pedh-CCK-like       1 ------------------------------------------------------------
Bacd-CCK-like       1 ------------------------------------------------------MFIYTR
Oocb-CCK-like       1 --------------------------------------------------MSIQ----GH
Drop-CCK-like      61 GSLPTFFLAYFSTPLCSSVSRRVASARLLQARADSSDQQGRIRGDPRDSLTIGTFYRYLR
Homos-CCKR1         1 ------------------------------------------------------------
Homos-CCKR2         1 ------------------------------------------------------------


Rhodniuspro-SKR1    1 ------------------------------------------------------------
Rhodniuspro-SKR2    1 ------------------------------------------------------------
Tricas-SKR1         7 NFTN-------------------NV-----------------------------------
Tricas-SKR2         1 ------------------------------------------------------------
Drom-SKR1           7 GDADQAAMAAAAAYRALLDYYANAPSAAGHIVSLNVAPYNG-TGNGGTVSLAGNA-----
Drom-SKR2          88 QALP----LAKEAAIHLNAS--------NEISA---------VGDGVTITGT--------
Anog-SKR           11 ELRD-------------------PPTAPTELTQ-----YDLLFGPGSLLYRPPN----SM
Pera-SKR           10 EMLD-------------------SEV--------------------NTSYN-------TS
Nill-CCK-like       1 ------------------------------------------------------------
Denp-CCK-like       1 ------------------------------------------------------------
Nicv-CCK-like       1 ------------------------------------------------------------
Athr-CCK-like       1 ------------------------------------------------------------
Neol-CCK-like       1 ------------------------------------------------------------
Pedh-CCK-like       1 ------------------------------------------------------M-----
Bacd-CCK-like       7 EYESEKFS--SYSYELKSKSTSPTPTYATGISVTSVEPAAA-LVAAGTL-----------
Oocb-CCK-like       7 NLSN-------------------NPV--------------------TTEYF-------GA
Drop-CCK-like     121 QLLSALAPLATDAVLQLNGT--------YAAQELYGGSPGG-LGGGGSGSGSGSGMAEQD
Homos-CCKR1         1 ------------------------------------------------------------
Homos-CCKR2         1 ------------------------------------------------------------
```

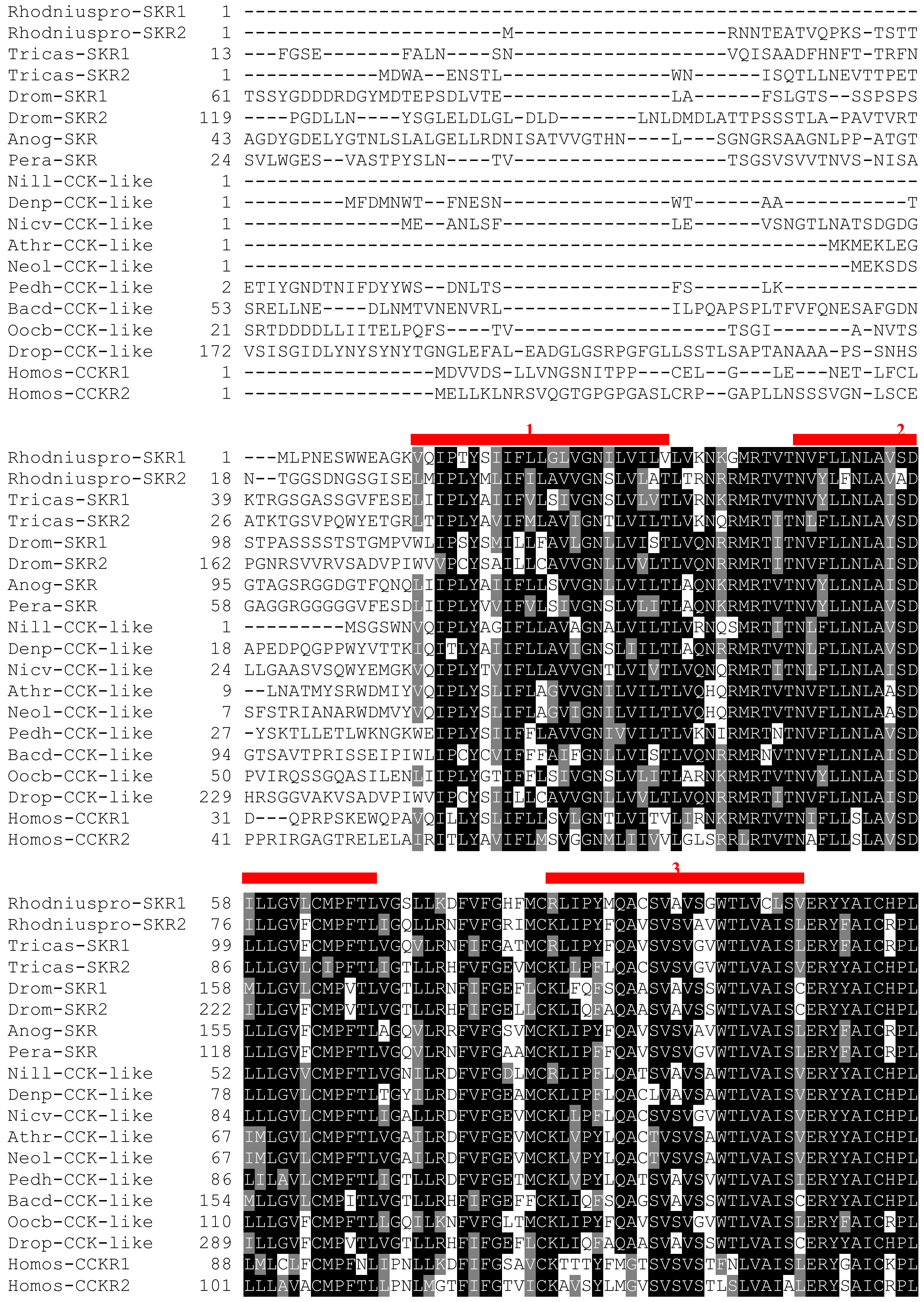
Rhodniuspro-SKR1 1 --
Rhodniuspro-SKR2 1 -----------------------M-------------------RNNTEATVQPKS-TSTT
Tricas-SKR1 13 ---FGSE-------FALN----SN-------------------VQISAADFHNFT-TRFN
Tricas-SKR2 1 ------------MDWA--ENSTL---------------WN------ISQTLLNEVTTPET
Drom-SKR1 61 TSSYGDDDRDGYMDTEPSDLVTE---------------LA------FSLGTS--SSPSPS
Drom-SKR2 119 ----PGDLLN----YSGLELDLGL-DLD-------LNLDMDLATTPSSSTLA-PAVTVRT
Anog-SKR 43 AGDYGDELYGTNLSLALGELLRDNISATVVGTHN----L---SGNGRSAAGNLPP-ATGT
Pera-SKR 24 SVLWGES--VASTPYSLN----TV-------------------TSGSVSVVTNVS-NISA
Nill-CCK-like 1 --
Denp-CCK-like 1 ---------MFDMNWT--FNESN---------------WT------AA-----------T
Nicv-CCK-like 1 --------------ME--ANLSF---------------LE------VSNGTLNATSDGDG
Athr-CCK-like 1 --MKMEKLEG
Neol-CCK-like 1 --MEKSDS
Pedh-CCK-like 2 ETIYGNDTNIFDYYWS--DNLTS---------------FS------LK------------
Bacd-CCK-like 53 SRELLNE----DLNMTVNENVRL---------------ILPQAPSPLTFVFQNESAFGDN
Oocb-CCK-like 21 SRTDDDDLLIITELPQFS----TV-------------------TSGI-------A-NVTS
Drop-CCK-like 172 VSISGIDLYNYSYNYTGNGLEFAL-EADGLGSRPGFGLLSSTLSAPTANAAA-PS-SNHS
Homos-CCKR1 1 -----------------MDVVDS-LLVNGSNITPP---CEL--G---LE---NET-LFCL
Homos-CCKR2 1 -----------------MELLKLNRSVQGTGPGPGASLCRP--GAPLLNSSSVGN-LSCE
1
2
Rhodniuspro-SKR1 1 ---MLPNESWWEAGKVQIPTYSIIFLLGLVGNILVILVLVKNKGMRTVTNVFLLNLAVSD
Rhodniuspro-SKR2 18 N--TGGSDNGSGISELMIPLYMLIFILAVVGNSLVLATLTRNRRMRTVTNVYLFNLAVAD
Tricas-SKR1 39 KTRGSGASSGVFESELIIPLYAIIFVLSIVGNSLVLVTLVRNKRMRTVTNVYLLNLAISD
Tricas-SKR2 26 ATKTGSVPQWYETGRLTIPLYAVIFMLAVIGNTLVILTLVKNQRMRTITNLFLLNLAVSD
Drom-SKR1 98 STPASSSSTSTGMPVWLIPSYSMILLFAVLGNLLVISTLVQNRRMRTITNVFLLNLAISD
Drom-SKR2 162 PGNRSVVRVSADVPIWVVPCYSAILLCAVVGNLLVVLTLVQNRRMRTITNVFLLNLAISD
Anog-SKR 95 GTAGSRGGDGTFQNQLIIPLYAIIFLLSVVGNLLVILTLAQNKRMRTVTNVYLLNLAISD
Pera-SKR 58 GAGGRGGGGGVFESDLIIPLYVVIFVLSIVGNSLVLITLAQNKRMRTVTNVYLLNLAVSD
Nill-CCK-like 1 ---------MSGSWNVQIPLYAGIFLLAVAGNALVILTLVRNQSMRTITNLFLLNLAVSD
Denp-CCK-like 18 APEDPQGPPWYVTTKIQITLYAIIFLLAVIGNSLIILTLAQNRRMRTVTNLFLLNLAVSD
Nicv-CCK-like 24 LLGAASVSQWYEMGKVQIPLYTVIFLLAVVGNTLVIVTLVQNQRMRTITNLFLLNLAISD
Athr-CCK-like 9 --LNATMYSRWDMIYVQIPLYSLIFLAGVVGNILVILTLVQHQRMRTVTNVFLLNLAASD
Neol-CCK-like 7 SFSTRIANARWDMVYVQIPLYSLIFLAGVIGNILVILTLVQHQRMRTVTNVFLLNLAASD
Pedh-CCK-like 27 -YSKTLLETLWKNGKWEIPLYSIIFFLAVVGNIVVILTLVKNIRMRTNTNVFLLNLAVSD
Bacd-CCK-like 94 GTSAVTPRISSEIPIWLIPCYCVIFFFAIFGNLLVISTLVQNRRMRNVTNVFLLNLAISD
Oocb-CCK-like 50 PVIRQSSGQASILENLIIPLYGTIFFLSIVGNSLVLITLARNKRMRTVTNVYLLNLAISD
Drop-CCK-like 229 HRSGGVAKVSADVPIWVIPCYSIILLCAVVGNLLVVLTLVQNRRMRTITNVFLLNLAISD
Homos-CCKR1 31 D---QPRPSKEWQPAVQILLYSLIFLLSVLGNTLVITVLIRNKRMRTVTNIFLLSLAVSD
Homos-CCKR2 41 PPRIRGAGTRELELAIRITLYAVIFLMSVGGNMLIIVVLGLSRRLRTVTNAFLLSLAVSD
3
Rhodniuspro-SKR1 58 ILLGVLCMPFTLVGSLLKDFVFGHFMCRLIPYMQACSVAVSGWTLVCLSVERYYAICHPL
Rhodniuspro-SKR2 76 ILLGVFCMPFTLIGQLLRNFVFGRIMCKLIPYFQAVSVSVAVWTLVAISLERYFAICRPL
Tricas-SKR1 99 LLLGVFCMPFTLVGQVLRNFIFGATMCRLIPYFQAVSVSVGVWTLVAISLERYFAICRPL
Tricas-SKR2 86 LLLGVLCIPFTLIGTLLRHFVFGEVMCKLLPFLQACSVSVGVWTLVAISVERYYAICHPL
Drom-SKR1 158 MLLGVLCMPVTLVGTLLRNFIFGEFLCKLFQFSQAASVAVSSWTLVAISCERYYAICHPL
Drom-SKR2 222 ILLGVFCMPVTLVGTLLRHFIFGELLCKLIQFAQAASVAVSSWTLVAISCERYYAICHPL
Anog-SKR 155 LLLGVFCMPFTLAGQVLRRFVFGSVMCKLIPYFQAVSVSVAVWTLVAISLERYFAICRPL
Pera-SKR 118 LLLGVFCMPFTLVGQVLRNFVFGAAMCKLIPFFQAVSVSVGVWTLVAISLERYFAICRPL
Nill-CCK-like 52 LLLGVVCMPFTLVGNILRDFVFGDLMCRLIPFLQATSVAVSAWTLVAISVERYYAICHPL
Denp-CCK-like 78 LLLGVLCMPFTLTGYILRDFVFGEAMCKLIPFLQACLVAVSAWTLVAISVERYYAICHPL
Nicv-CCK-like 84 LLLGVLCMPFTLIGALLRDFVFGEVMCKLLPFLQACSVSVGVWTLVAISVERYYAICHPL
Athr-CCK-like 67 IMLGVLCMPFTLVGAILRDFVFGEVMCKLVPYLQACTVSVSAWTLVAISVERYYAICHPL
Neol-CCK-like 67 IMLGVLCMPFTLVGAILRDFVFGEVMCKLVPYLQACTVSVSAWTLVAISVERYYAICHPL
Pedh-CCK-like 86 LILAVLCMPFTLIGTLLRDFVFGETMCKLVPYLQATSVAVSVWTLVAISIERYYAICHPL
Bacd-CCK-like 154 MLLGVLCMPITLVGTLLRHFIFGEFFCKLIQFSQAGSVAVSSWTLVAISCERYYAICHPL
Oocb-CCK-like 110 LLLGVFCMPFTLLGQILKNFVFGLTMCKLIPYFQAVSVSVGVWTLVAISLERYFAICRPL
Drop-CCK-like 289 ILLGVFCMPVTLVGTLLRHFIFGEFLCKLIQFAQAASVAVSSWTLVAISCERYYAICHPL
Homos-CCKR1 88 LMLCLFCMPFNLIPNLLKDFIFGSAVCKTTTYFMGTSVSVSTFNLVAISLERYGAICKPL
Homos-CCKR2 101 LLLAVACMPFTLLPNLMGTFIFGTVICKAVSYLMGVSVSVSTLSLVAIALERYSAICRPL

```
                                                4
Rhodniuspro-SKR1 118 RSRTWQTLTHAYRLIGAIWVCSLLLMTPISVLSELIPTS-G---GHRKCRELWPN--EDI
Rhodniuspro-SKR2 136 KSRRWQTQFHAYKMIAIVWAMSLVWNSPILFVSRLLAMG-GK--GRHKCREVWPG--RRS
Tricas-SKR1      159 KSRRWQTQFHAYKMIAVVWLASLFWSAPVLAVSSLKAMK-GR---GHKCREEWPS--KSS
Tricas-SKR2      146 RSLRWQTISHAYKLIVGIWIGSLICMAPIALLSQLKPTK-Q---GNYKCREDWPS--LDY
Drom-SKR1        218 RSRSWQTISHAYKIIGFIWLGGILCMTPIAVFSQLIPTS-RP--GYCKCREFWPD--QGY
Drom-SKR2        282 RSRTWQTINHANKIIAIIWLGSLVCMTPIAAFSQLMPTS-RP--GLRKCREQWPADSLNY
Anog-SKR         215 SSRRWQTQFHAYKMIGLVWTVSFLANSPLGYVQRLLPVGRST--GQMKCREEWPS--PAW
Pera-SKR         178 KSRRWQTQFHAYKTIVVVWLASLIWNSPIFVVSRLQAIK-ET--ERHKCREDWPS--KSS
Nill-CCK-like    112 RSRRWQTLSHAYRLIAVIWLASFVTMLPISLLSRLIPTN-Q---GLKKCREIWPD--ADY
Denp-CCK-like    138 RSLAWQTLSHAYKTILGIWICSFICMLPIAFLSELKPTI-K---GNRKCRENWAS--LEY
Nicv-CCK-like    144 RSLRWQTISHAYKLIIAIWAGSVICMGPIALLSQLKPTN-Q---GNHKCREDWHT--LDY
Athr-CCK-like    127 RSRRWQTLSHSYRLIAVIWTGSLLLMSPIAVLSELKPTS-N---GHHKCRENWPGH-GEY
Neol-CCK-like    127 RSRRWQTLSHSYRLIAVIWTGSLILMSPIAVLSELKPTS-N---GHQKCRENWPGH-GEY
Pedh-CCK-like    146 RSRRWQTLSHAYHLICYIWTGSTITMLPIFILTELQPTN-K---GRHKCREKWPN--GDY
Bacd-CCK-like    214 RSRTWQTINHAYRIIGFIWFGSIICMTPIALFSQLIPTS-RQ--GLRKCRDQWPEDTIAY
Oocb-CCK-like    170 KSRRWQTQFHAYKMIAVVWTLSLTWNMPILVVSRLKSLR-G---GRRKCREEWPS--VGS
Drop-CCK-like    349 RSRTWQTINHANKIIAIIWLGSLLCMTPIALFSQLMPTS-RP--GLRKCREQWPADSLNY
Homos-CCKR1      148 QSRVWQTKSHALKVIAATWCLSFTIMTPYPIYSNLVPFTKNNNQTANMCRFLLPN--DVM
Homos-CCKR2      161 QARVWQTRSHPARVIVATWLLSGLLMVPYPVYTVVQPVGPR----VLQCVHRWPS--ARV

                                            5
Rhodniuspro-SKR1 172 EKTYNLLLDFLLLVIPLIVMVTTYTLVAKTLWRVMKTQKPGNEMGLKD------------
Rhodniuspro-SKR2 191 EGAYIIFLDIVLLMIPLLIMSLAYSLIVLKLWKGLQRELKHSNSCLQTVDR-SASLPTMT
Tricas-SKR1      213 EQIFNLFLDAMLLLIPVLIMSLAYSLIMTKLWKGLRREIQHNNSFQAQMIQRSNSSPTIN
Tricas-SKR2      200 EKAYNLFLDVVLLVIPLLVLGVTYSLITRTLCKGMKTERALRDHTVNGAVDVYINL----
Drom-SKR1        273 ELFYNILLDFLLLVLPLLVLCVAYILITRTLYVGMAKDSGRILQQSLPVSATTAGG-SAP
Drom-SKR2        339 ERAYNLFLDLALLVLPLLALSFTYLFITRTLYVSMRNERAMNFGSSGPEVTTSSSA-AVA
Anog-SKR         271 EKAYVLFHDVGLLFLPLLTMGFAYSMIVSKLWRGLRHEIKHSSLYQQTSRQHGTGGQGSS
Pera-SKR         233 ERAYNIFLDAMLLLVPLIIMTLAYSLIVSKLWKGLRREIRHNSSCRRQLER-TSSSATVN
Nill-CCK-like    166 ERGYNLMLDAVLLVLPLFILIVTYSLITITLWRGIRPSNQSGQGELMNNGCSHIEV----
Denp-CCK-like    192 EKAFNLVLDIVLMVFPLLVLAATYSLIIRTLWKGIKTERASRSTGSTM--EIYVNL----
Nicv-CCK-like    198 EKAFNIFLDVVLLVVPLFVLAVTYSLITNTLCKGMQTERTLRDSGAGGVVEVYINM----
Athr-CCK-like    182 EKAYNLILDAVLLIFPLVILVTTYTLITIPLQSSVDPAVVRHSANGSVVSEVYLGS----
Neol-CCK-like    182 EKAYNLILDAVLLIFPLIILVTTYTLITITLQSSVDPT-------------VYLGS----
Pedh-CCK-like    200 ERIYNLLLDLLLLLIPLLALGVTYYLISITLWRDLSKKSEDVSYQRHEENSKREKN----
Bacd-CCK-like    271 ERFYNIFLDLTLLVLPLFVLCVAYILITRTLYVGMRAERALVLGGNSTNGSTPAKQ----
Oocb-CCK-like    224 ERAYNLFLDGTLLLVPLLLMSLAYSLIAIKLWRGLKLEIRQSSTRTKRHLS---------
Drop-CCK-like    406 ERAYNLFLDLALLVLPLLALSFTYLFITRTLYVSMRNERAMNFGSSGPE-----------
Homos-CCKR1      206 QQSWHTFLLLILFLIPGIVMMVAYGLISLELYQGIKFEASQKKSAKERKP----------
Homos-CCKR2      215 RQTWSVLLLLLLFFIPGVVMAVAYGLISRELYLGVRFDGDSDSDSQSRVRNQG-GL---P

Rhodniuspro-SKR1 220 ------------------------------------------------------------
Rhodniuspro-SKR2 250 EVVISKN-LNTN-SEAI---------------HRVI---------------------PAD
Tricas-SKR1      273 GELNKSTSPQSTSEPSGMN-HLRPT-------NRLL-------------PPSHNKAHSRV
Tricas-SKR2      256 -HG---S-------------STT-----------------SRWSK---------------
Drom-SKR1        332 NPG---T---SSSSNCILVLTAT-----------------AVYNENSN------------
Drom-SKR2        398 EAG---SQRRANGSHCQSLDTIVPHQHNPHQQHHHHSQYYYDYGHCGSKRRLISGGGPCE
Anog-SKR         331 -VG---GAPTG--AAAGSV-GT--------------------------------ASGEQHCT
Pera-SKR         292 EVVMATSNINGN-STTGSA-TITPL-----RQHRLLKQQQM------LNRQSAGSTTLCA
Nill-CCK-like    222 -TR---S-------------SSS-----------------FK------------------
Denp-CCK-like    246 -QM---N-------------SRT-----------------SWRIKNK-------------
Nicv-CCK-like    254 -QS---T-------------SS------------------KRWSR---------------
Athr-CCK-like    238 -RG---D-------------TLT-----------------R----CSL------------
Neol-CCK-like    225 -RG---D-------------TLT-----------------R----CSL------------
Pedh-CCK-like    256 -SS---Y-------------DLS-----------------DEYNPNNN------------
Bacd-CCK-like    327 --H---D-----------------------------------------------------
Oocb-CCK-like    275 -------------------------------------QGPR------IQI----------
Drop-CCK-like    455 ------------------------------------------------------------
Homos-CCKR1      256 ----------------------------------------------S-------------
Homos-CCKR2      271 GAV------HQ-----------------------------NGRCR---------------
```

```
Rhodniuspro-SKR1 220 ------------------------------------------------------------
Rhodniuspro-SKR2 272 SQQGQFCNKNPVQMWLLKVKLEE-------------------------------------
Tricas-SKR1      312 KDAKHSKKTESVKMWFMKGIVQVRLPASIKKGYTCNTQTKSTLVPRCELTT---PSS-EH
Tricas-SKR2      267 ------------------------------------------------------------
Drom-SKR1        357 ------------------------------------------------------------
Drom-SKR2        455 GRRHLYC---------MRS---------------------------ASVK----------
Anog-SKR         354 GDTGKPPKPPGG-----------------------KRHEPG-FVLTSSLKKAPFKSSTAV
Pera-SKR         339 GRDGNNRAATSVKLWLFKGMVQVRLPSSSSNAQSRYRERPGSLSSSKSVTIAPLPTATSA
Nill-CCK-like    230 ------------------------------------------------------------
Denp-CCK-like    259 ------------------------------------------------------------
Nicv-CCK-like    264 ------------------------------------------------------------
Athr-CCK-like    248 ------------------------------------------------------------
Neol-CCK-like    235 ------------------------------------------------------------
Pedh-CCK-like    270 ------------------------------------------------------------
Bacd-CCK-like    329 ------------------------------------------------------------
Oocb-CCK-like    282 ------------------------------------------------------------
Drop-CCK-like    455 ------------------------------------------------------------
Homos-CCKR1      257 --------TTSS------------------------------------------------
Homos-CCKR2      281 --------PETG------------------------------------------------

Rhodniuspro-SKR1 220 ----------------------------------------------NRV-T-W--KQN--
Rhodniuspro-SKR2 295 -----------------------------GIAQR----------------------SGTP
Tricas-SKR1      368 CSY--------------------------NELCPS--------------------NDASY
Tricas-SKR2      267 --------------------------------------RHNPNWRQLRH-N-W--SQESS
Drom-SKR1        357 ---------------NNNGNSEGSAGGGSTNMATTTLTTRPTAPTVITT-T-T-TTTVTL
Drom-SKR2        469 --------SLRHQQINGGGGTLSGTGAGNGECCS-RVHRMR-QQMQLQQQGYVSDNESRR
Anog-SKR         390 VNFASNNNTINKSNFSGAG--SGSSSNGAGSSGSS---NGS--------NGGGCNGSGAD
Pera-SKR         399 GTYNTS---PETSRLIG-------SSNGNGAACRDDKDLGG--------AGATVDGEDVN
Nill-CCK-like    230 ---------------------------------------------------------FNS
Denp-CCK-like    259 ---------------TN-------------------MSRQQSNWTT-LR-N-W--SDESN
Nicv-CCK-like    264 -------------------------------------TSRGTSLRQLRQ-N-W--SQDST
Athr-CCK-like    248 ---------------RT-------------------LERTPSSRRSLRL-V-TKPSLNAV
Neol-CCK-like    235 ---------------RT-------------------LDRTPSSRRSLRL-V-AKSSVGGM
Pedh-CCK-like    270 ---------------NN-------------------IT-TGRNKKKLKR-E-IILNDVTK
Bacd-CCK-like    329 ----------------------------------------NSSL-------------GAI
Oocb-CCK-like    282 ------------------------RANGSGCLTRETKDNSN--------E----D-Q---
Drop-CCK-like    455 ------------------------------------------------------------
Homos-CCKR1      261 ------------------------GKYEDSDGCYLQKTRPP---RKLEL-----------
Homos-CCKR2      285 ------------------------AVGEDSDGCYVQLPRSR---PALEL-----------

                                                                    6
Rhodniuspro-SKR1 228 -----------SRGSPHLRRSNTEKALKKKKRVVKMLFAVVLEFFVCWTPLYVINTITLF
Rhodniuspro-SKR2 304 LEPL-ESPGPK-FTRHAIRSNYMDKSIEAKKKVIRMLFVVVAEFFICWAPLHVLNTWYQF
Tricas-SKR1      382 ATSSVDETTYH-FTRHAIRSNYMDKSIEAKKKVIRMLFVVVAEFFICWAPLHILNTWYLF
Tricas-SKR2      285 S-PGTGS----QKYTPGLRRTNAERSLLNKKRVIKMLFAVVLEFFICWTPLYVINTIVLF
Drom-SKR1        399 A-KTSSP--SIRVHDAALRRSNEAKTLESKKRVVKMLFVLVLEFFICWTPLYVINTMVML
Drom-SKR2        519 K-SLSQP--SLRITEAGLRRSNETKSLESKKRVVKMLFVLVLEFFICWTPLYVINTMTML
Anog-SKR         437 Q-----RTHYCGGAGCETRPGRLRGFRHFFAKVIRMLFVIIVEFFVCWAPLHILNTVYLY
Pera-SKR         441 IPPGESPNTYT-FTRHAIRSNYMDKSIEAKKKVIRMLFVVVAEFFVCWAPLHVLNTWYLF
Nill-CCK-like    233 T-VGTTS----SSSPGGLRRTNTEKALLKKKRVIKMLCAVVLEFFICWTPLYVINTVTLF
Denp-CCK-like    280 S-PVGSN--SSKRLVSGLRRTNAERSLCNKKRVIKMLCVVVLEFFVCWSPLYTVNTIVLF
Nicv-CCK-like    283 S-PIGS-------QRTGLRRSNAERSLTNKKRVIKMLFAVVLEFFICWTPLYVINTIVLF
Athr-CCK-like    272 K-PWDES----DLSQHTTRGRSALLKRKRKRRIVEMLCVVVLEFFLFWTPLYVINTVALF
Neol-CCK-like    259 K-PWDDQ---GEASQRSTRGRSAMLKRKRKRRIVEMLCVVVLEFFLFWTPLYVINTVALF
Pedh-CCK-like    293 L-EVFQS--IKSCSSISLRHNNYERSLRKKQLVIKMLFVVVLEFFVCWTPLYVINTLALF
Bacd-CCK-like    336 A-TV-TK--STAAAPSATLQSNEAKNLESKKRVVKMLFVLVLEFFICWTPLYVINTISMF
Oocb-CCK-like    302 ----QNSICPL-SRQHVIRSNYMGKSIEAKKKVIRMLFVIVLEFFVCWAPLHVINTWYLF
Drop-CCK-like    455 ----------------------------SKKRVVKMLFVLVLEFFICWTPLYVINTMTML
Homos-CCKR1      283 ----RQLSTGSSSRANRIRSNSSAANLMAKKRVIRMLIVIVVLFFLCWMPIFSANAWRAY
Homos-CCKR2      307 ----TALTAPGPGS----GSRPTQAKLLAKKRVVRMLLVIVVLFFLCWLPVYSANTWRAF
```

7

```
Rhodniuspro-SKR1 277 APQAVYE-RLGYKGISFLQLLAYSSSCCNPITYCFMNYRFRRAFLKLFG--CLREEKGSS
Rhodniuspro-SKR2 362 RPDLVHQ-YVGSTGVSLVQLLAYISSCCNPITYCFMNYRFRQAFISLFNFPR----LCC-
Tricas-SKR1      441 YPEDVYL-YVGSTGISLVQLLAYISSCCNPITYCFMNRKFRQAFLAIFNWYNVC--YCC-
Tricas-SKR2      340 DSSVIYN-NIGYKAITFFQLLAYCSSCCNPITYCFMNCGFRKSFLNLFK--CLKKSRN--
Drom-SKR1        456 IGPVVYE-YVDYTAISFLQLLAYSSSCCNPITYCFMNASFRRAFVDTFKGLPWRRGAGAS
Drom-SKR2        576 LGPTVYE-YVGYTSISFLQLLAYSSSCCNPITYCFMNASFRRAFVDTFKGMRVCERLCA-
Anog-SKR         492 SPTFVYQ-YVNSSGIALVQLMAYISSCCNPITYCFMNRRFRQAFLGVFSCYRNRMPICCC
Pera-SKR         500 NPEAVYS-TVGSTGVSLVQLLAYISSCCNPITYCFMNRKFRQAFLGVFDCYRCWSMYCCC
Nill-CCK-like    288 DKYIVYN-ALSFQAISFFQLLAYSSSCCNPITYCFMNSSFRKAFLKLFG--CLRRGRPKT
Denp-CCK-like    337 TGQYVYD-TIGYTGISLLQLLAYTSSCCNPITYCFMNGGFRKSFLKLFS--CFKRSRYRA
Nicv-CCK-like    335 DAEIVYN-NLGYTVIGYLQLLAYSSSCCNPITYCFMNCGFRKSFMKLFR--CHREAQSNK
Athr-CCK-like    327 NPDALYD-GLTPTWISYFHLLAFCSSCCNPITYCFMSSGFRKSFSGLFA--CCRDCGYRR
Neol-CCK-like    315 SPDALYD-GLNPTWISYFHLLAFCSSCCNPITYCFMSSGFRKSFSGLFTGCCSRACGCRK
Pedh-CCK-like    350 WPHLVYRGKIGYKMISFCQLLAYTSGCCNPITYCFMNKGFRTWKKKKEQ-----------
Bacd-CCK-like    392 IGPAIYE-YIDYTSICFFQLLAYSSSCCNPITYCFMNASFRRAFLDTFKGLRMSSVTSRR
Oocb-CCK-like    357 APDLVYS-VVGSTGISLVQLLAYVSSCCNPITYCFMNKKFRQAFLGLFDCHRCWRVSCRH
Drop-CCK-like    487 IGPVVYE-YVDYTAISFLQLLAYSSSCCNPITYCFMNASFRRAFVDTFKV-RMCD--GG-
Homos-CCKR1      339 DTASAER-RLSGTPISFILLLSYTSSCVNPIIYCFMNKRFRLGFMATF-PCCPNPGPPGA
Homos-CCKR2      359 DGPGAHR-ALSGAPISFIHLLSYASACVNPLVYCFMHRRFRQACLETCARCCPRPPRARP

Rhodniuspro-SKR1 334 ------------------------------------------------------------
Rhodniuspro-SKR2 416 WCGIPVES----KLA-------------QRTDTANEPNSLSANDSTLYAGRAN---RSEV
Tricas-SKR1      497 VCMEPKSH----R-TRTKTAQKNGI-----HKIIQNNSDVSCNESTIYIGRQSTIGRS-V
Tricas-SKR2      395 F-------GVTGSEINMETKWTN-R--------CSENAD---------------------
Drom-SKR1        515 G-------GVGGA-AGGGLSASQAG--------AGPGAYASANTNIS------LNPGL-A
Drom-SKR2        634 -------------PCCFWRRRSKNE------------------TNLSVAGNSIALANS-V
Anog-SKR         551 FCCASSNGPMEGAESKAAAYLRQNTI--NQSGAERNNSDMSGNDSLVYVGRGLVQRSG-K
Pera-SKR         559 CCGDVVAL----RSSRLGGLQRDGAA--LGGLGANNNSDVSGNDSTVFAGRASVGARSEV
Nill-CCK-like    345 H-------F---------------------------------------------------
Denp-CCK-like    394 N------FRMEGSDCQVDLKYSNQR--------CSEN-----------------------
Nicv-CCK-like    392 S-------LLNGSEFNMDTKCAI-R--------NEPL-----------------------
Athr-CCK-like    384 S--------GGG-RYR-----------------EPP------------------------
Neol-CCK-like    374 R--------AEG-RYR-----------------EPP------------------------
Pedh-CCK-like        ------------------------------------------------------------
Bacd-CCK-like    451 V---------GEAGFSGWVKRRRRTTGGGDSVSCTQLPALGTNNSISLTNNTIIVPNI-N
Oocb-CCK-like    416 ND--V-AM----ATTA-------------NAGQTGNNSELSGNDSAMYLGRASLVARSGI
Drop-CCK-like    542 -------------RLCFWRRRSKNE------------------TNLSVAGNSIALANS-V
Homos-CCKR1      397 R----------G-----EV--GEE---------EEGGTTGASLSRFSYSHMSASVPPQ--
Homos-CCKR2      418 R----------------AL--PDE---------DPPTPSIASLSRLSYTTISTLGPG---

Rhodniuspro-SKR1     -------------------------------------
Rhodniuspro-SKR2 456 MVLEKEERV----------------------------
Tricas-SKR1      546 VVLEAEDRV----------------------------
Tricas-SKR2      418 ----------------FCR------------------
Drom-SKR1        552 M---GMGTWRSRSRHEFLNAVVTTNSAAAAVNSPQL-
Drom-SKR2        662 M---SSHTI---------------------LESPRL-
Anog-SKR         608 LCARRSGTYPRYYRDAVCNALPWDLKM----------
Pera-SKR         613 VVLEAEDRV----------------------------
Nill-CCK-like        -------------------------------------
Denp-CCK-like        -------------------------------------
Nicv-CCK-like        -------------------------------------
Athr-CCK-like        -------------------------------------
Neol-CCK-like        -------------------------------------
Pedh-CCK-like        -------------------------------------
Bacd-CCK-like    501 T---AMHN---------------------SQTPQSN
Oocb-CCK-like    456 IILLN--------------------------------
Drop-CCK-like    570 M---SSHTI---------------------LESPRL-
Homos-CCKR1          -------------------------------------
Homos-CCKR2          -------------------------------------
```

Supplementary Figure S4. Luminescence response of forskolin and dibutyryl cAMP (dbcAMP) for Rhopr-SKR-1 transiently expressed in HEK293/CNG cells. Figure displays kinetics of response activation at 5-second intervals for a total of 30 seconds.

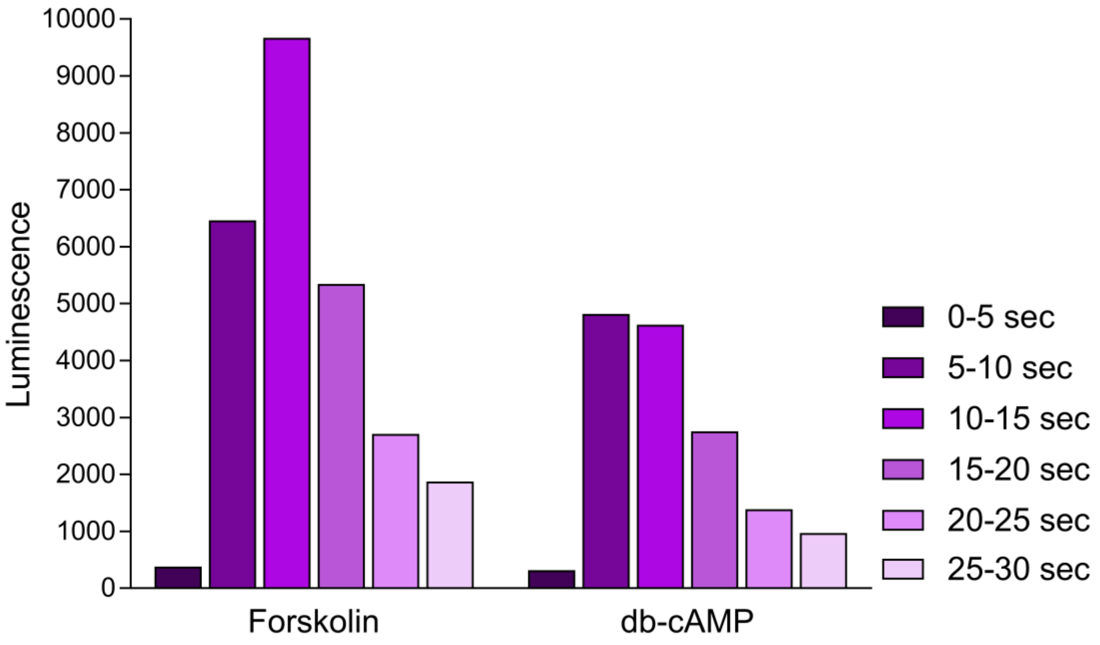

Luminescence
10000
9000
8000
7000
6000
5000
4000
3000
2000
1000
0
Forskolin
db-cAMP
0-5 sec
5-10 sec
10-15 sec
15-20 sec
20-25 sec
25-30 sec

Supplementary Tables

Supplementary Table S1. Primer sequences utilized to generate the complete Rhopr-SKR-1 and Rhopr-SKR-2 sequences (5’ to 3’).

**pDNR (Containing cDNA Library) Plasmid-Specific Primers**	
pDNR-For1	GTGGATAACCGTATTACCGCC
pDNR-For2	ACGGTACCGGACATATGCC
pDNR-Rev25	GTGGATAACCGTATTACCGCC
**Rhopr-SKR-1 5’ Region Amplification**	
SKR1-Rev1	CGAAATCTTTTAGTAAGGATCCA
SKR1-Rev2	ACACATTTGTTACAGTTCTCATTCC
SKR1-Rev3	CCAGGCCAAGTAAAAAGATGATT
**Rhopr-SKR-1 3’ Region Amplification**	
SKR1-For1	GAAGCTGGTAAAGTACAAATTCC
SKR1-For2	GCAGTTTCTGACATATTACTTGG
SKR1-For3	CTAAAAGATTTCGTGTTTGGAC
**Rhopr-SKR-2 5’ Region Amplification**	
SKR2-For1	TGGTAACAGTGCGCATTCT
SKR2-For2	GCCAAAACTAAGGAATTACCAAC
SKR2-For3	CCACTGCCATTATCACTGC
**Rhopr-SKR-2 3’ Region Amplification**	
SKR2-Rev1	TGTCTTTAGTGTGGAATTCGC
SKR2-Rev2	AGCGAAGGAGCGTATATAATATTTC
SKR2-Rev3	GTCCTGTTGATGATTCCGTTAT

Supplementary Table S2. Primers used for the insertion of Rhopr-SKR-1 and Rhopr-SKR-2 into the mammalian expression vector pIRES2-ZsGreen1 with the restriction sites bolded and the kozak sequences underlined (5' to 3').

**Rhopr-SKR-1**	
Rhopr-SKR-1 Fwd	TACGCA**GAGCTC**GCCACCATGTTGCCAAATGAATCTTG
Rhopr-SKR-1 Rvrs	TCAGCTTGATCCTTTTTCTTC**CCGCGG**TTATTA
**Rhopr-SKR-2**	
Rhopr-SKR-2 Fwd	ATTATA**GAGCTC**GCCACCATGCGGAACAACACTGAA
Rhopr-SKR-2 Rvrs	CTAAACTCGCTCTTCCTTTTCTA**CCGCGG**TTATTA

Supplementary Table S3. Primer sequences utilized to measure spatial distribution of the transcripts for the Rhopr-SKRs and Rhopr-SKs via Reverse transcriptase quantitative PCR (RT-qPCR), as well as those utilized for the housekeeping genes (5' to 3').

**Rhopr-SKR-1**	
Rhopr-SKR-1 qPCR Fwd	GAAGCTGGTAAAGTACAAATTC
Rhopr-SKR-1 qPCR Rvrs	CGAAATCTTTTAGTAAGGATCCA
**Rhopr-SKR-2**	
Rhopr-SKR-2 qPCR Fwd	TCCAAGCTGTTTCAGTATCAGTAG
Rhopr-SKR-2 qPCR Rvrs	GCGAATTCCACACTAAAGACA
**Rhopr-Housekeeping Genes**	
α-TUB Fwd	GTGTTTGTTGATTTGGAACCTACAG
α-TUB Rvrs	CCGTAATCAACAGACAATCTTTCC
β-Actin Fwd	AGAGAAAAGATGACGCAGATAATGT
β-Actin Rvrs	ATATCCCTAACAATTTCACGTTCG
Rp-49 Fwd	GTGAAACTCAGGAGAAATTGGC
Rp-49 Rvrs	AGGACACACCATGCGCTATC
**Rhopr-SK (peptides' transcript)**	
Rhopr-SK qPCR Fwd	AGCCAGCTGAAAGGAGATCA
Rhopr-SK qPCR Rvrs	GGTAAAGAATTGGCCATGGTCT

Supplementary Table S4. Primer sequences utilized to generate double stranded RNA templates of Rhopr-SKR-1, Rhopr-SKR-2, and Rhopr-SK, or the ampicillin resistance gene (ARG) (5' to 3').

**Rhopr-SKR-1**	
T7 Rhopr-SKR-1 Fwd	**TAATACGACTCACTATAGGGAGAGA**AGCTGGTAAAGTACAAATTC
Rhopr-SKR-1 Rvrs	CGAAATCTTTTAGTAAGGATCCA
Rhopr-SKR-1 Fwd	GAAGCTGGTAAAGTACAAATTC
T7 Rhopr-SKR-1 Rvrs	**TAATACGACTCACTATAGGGAGA**CGAAATCTTTTAGTAAGGATCCA
**Rhopr-SKR-2**	
T7 Rhopr-SKR-2 Fwd	**TAATACGACTCACTATAGGGAGA**TCCAAGCTGTTTCAGTATCAGTAG
Rhopr-SKR-2 Rvrs	GCGAATTCCACACTAAAGACA
Rhopr-SKR-2 Fwd	TCCAAGCTGTTTCAGTATCAGTAG
T7 Rhopr-SKR-2 Rvrs	**TAATACGACTCACTATAGGGAGA**GCGAATTCCACACTAAAGACA
**Rhopr-SK (SK peptides)**	
T7 Rhopr-SK Fwd	**TAATACGACTCACTATAGGGAGA**ATAATGGGTAGCAGCTTCC
Rhopr-SK Rvrs	GCATACACTCCACTTATAATTCCTCTAATTTAT
Rhopr-SK Fwd	**TAATACGACTCACTATAGGGAGA**GCATACACTCCACTTATAATTCC
T7 Rhopr-SK Rvrs	ATAATGGGTAGCAGCTTCCTAATCAC
